# Improving polygenic risk score based drug response prediction using transfer learning

**DOI:** 10.1038/s41525-025-00528-x

**Published:** 2025-11-21

**Authors:** Youshu Cheng, Song Zhai, Wujuan Zhong, Rachel Marceau West, Judong Shen

**Affiliations:** 1 Department of Biostatistics, Yale University, New Haven, CT USA; 2 Biostatistics and Research Decision Sciences, Merck & Co., Inc., Rahway, NJ USA

**Keywords:** Computational biology and bioinformatics, Drug discovery, Genetics, Biomarkers, Diseases, Medical research, Molecular medicine, Risk factors

## Abstract

Traditional methods for pharmacogenomics (PGx), like those using disease-specific polygenic risk scores (PRS-Dis), often fail to capture the full heritability of drug response, leading to poor predictions. Direct PGx PRS approaches could improve this, but the scarcity of relevant PGx datasets limits the wide application. To overcome these challenges, we introduce PRS-PGx-TL, a novel transfer learning method. It models large-scale disease summary statistics data alongside individual-level PGx data, leveraging both sources to create more accurate prognostic and predictive polygenic risk scores. In PRS-PGx-TL, we further develop a two-dimensional penalized gradient descent algorithm that starts with weights from disease data and then optimizes them using cross-validation. In simulations and an application to IMPROVE-IT (ClinicalTrials.gov, NCT00202878, September 13, 2005) PGx GWAS data, PRS-PGx-TL significantly enhances prediction accuracy and patient stratification compared to traditional PRS-Dis methods. Our approach shows great promise for advancing precision medicine by using an individual’s genetic information to guide treatment decisions more effectively.

## Introduction

Polygenic risk scores (PRSs) have recently emerged as promising tools in disease genome-wide association studies (GWAS) for predicting human diseases and complex traits^1–3^. A PRS combines multiple single-nucleotide polymorphisms (SNPs) into a single aggregated score that can be used to predict disease risk. It is an individual-level score calculated based on the number of risk variants that a person carries, weighted by SNP effect sizes that are derived from an independent large-scale disease GWAS summary statistics data. Similarly, PRS methods have also been recently applied to drug response prediction in pharmacogenomics (PGx) GWAS from randomized clinical trials (RCTs)^4–8^. However, it is generally more challenging in the PGx (and drug response) setting than the disease setting since PGx GWAS data from RCTs are usually from two arms (treatment and control), and both prognostic and predictive effects are used to construct PRS for drug response prediction. The prognostic effect measures the genotype (G) main association strength with the clinical outcome regardless of treatment, while the predictive effect measures the genotype-by-treatment (G × T) interaction association strength with the clinical outcome after treatment.

There are three main strategies to construct PRS in PGx GWAS for drug response prediction and patient stratification. The first strategy is to leverage disease GWAS summary statistics derived from large-scale and well-studied GWAS of related disease phenotypes, which assumes causal variants are shared across disease cohort and drug response (or PGx) population. The rationale behind this strategy is that genetic variations such as SNPs are increasingly recognized for their pleiotropic effects, influencing both an individual’s susceptibility to disease and their response to pharmacotherapy. A prime example is the APOE gene, where specific alleles like the isoform APOE4 are associated with an increase in low-density lipoprotein cholesterol (LDL-C) levels^9^, a well-known cardiovascular disease risk factor, and also influence an individual’s lipid-lowering response to statin drugs^10^. Similarly, variants in the CYP2C9 gene not only dictate the specific warfarin dose required to achieve therapeutic anticoagulation^11^ but also influence an individual’s risk of bleeding complications when on warfarin therapy, largely due to their impact on drug metabolism^11,12^. However, our previous research shows that this Disease PRS approach (PRS-Dis) lacks the ability to incorporate any predictive (or genotype-by-treatment interaction) effects in the PRS training stage and thus cannot fully capture the heritability of drug response^4,5^. In other words, directly applying disease PRS to PGx studies in the target cohort might not fully recover the heritability of drug response since it relies on a stringent assumption (the genotype-by-treatment interaction effect is proportional to the main genotype effect for every causal variant), which is barely satisfied in real PGx data^4,5^. This is because in PGx studies, a patient’s clinical outcomes are influenced by both prognostic and predictive effects, but a disease PRS is built with prognostic information only.

The second strategy is to acknowledge the difference between disease and drug response phenotypes as well as between their underlying genetic architectures and construct PRS based on only drug response or PGx-related variants. This strategy leverages PGx GWAS summary statistics derived from an independent PGx study of related drug response phenotype, so-called a direct PGx PRS approach (PRS-PGx), which has the potential to significantly improve the PRS-based drug response prediction performance^4,5^. Zhai et al. propose a series of PRS-PGx methods using PGx GWAS summary statistics or individual-level data^4^. However, such PRS-PGx approach has its own limitations, primarily due to (1) the much smaller sample sizes in PGx GWAS data in the base and target cohorts from RCTs (while compared with large disease cohorts), which may be insufficient for accurate estimation and prediction during PRS construction, especially in the base cohort; (2) limited availability of an independent PGx GWAS summary statistics or individual data^5^.

The third strategy is to leverage both large-scale disease GWAS summary statistics in the base (training) cohort and individual-level PGx data in the target cohort and jointly model them for learning parameters, constructing PRS and predicting drug response in the target cohort. Zhai et al. explore this strategy by simply replacing prognostic effects from PGx GWAS with prognostic effects from disease GWAS by assuming they are the same^5^. The authors demonstrate the potential of leveraging both disease and PGx GWAS data to improve the drug response prediction accuracy in most scenarios. But such a simple approach might not always increase the prediction accuracy in some special scenarios (i.e., when the sample size of a PGx GWAS data is large enough) since it does not consider the genetic (correlation) relationship between a drug response phenotype (in the target cohort) and a relevant disease phenotype (in the base cohort), and the assumption that the prognostic effects in disease and PGx studies are the same may not be true in most cases. Therefore, due to potential differences in the genetic architectures between drug response and disease phenotype, it is desired to jointly model and integrate disease and PGx GWAS data in a more efficient way to tackle this cross-phenotype (i.e., between disease phenotype and drug response phenotype) or cross-population (i.e., between disease population and PGx population) prediction challenge.

Traditional PRS methods often struggle when sample sizes are limited or when there is population heterogeneity; these challenges are frequently encountered in underrepresented populations (i.e., non-European populations), where it is difficult or costly to obtain a larger sample size (e.g., PGx population). In such cases, Transfer Learning (TL) provides a solution by leveraging knowledge from a large and well-studied (base) population to improve PRS prediction of a related phenotype in a smaller and less-studied (target) population. TL is a machine learning technique where a model trained on one task is repurposed on a second related task, resulting in increased efficiency by utilizing the connectivity between the prior training samples and related testing data^13–15^.

In the field of PRS research, a few TL-based methods have been recently developed to construct more powerful PRS for cross-population prediction in trans-ethnic disease GWAS by applying a baseline model pre-trained with GWAS summary statistics from an ancestry group of a larger sample size to a smaller target ancestry group^4,16,17^. Zhao et al.^4^ develop a transfer learning-based PRS method called TL-PRS to fine-tune a baseline PRS model (built from the European population) to a target ancestry (South Asian or African ancestry), which demonstrates its ability of improving the transferability of PRS across different ancestries. Jeng et al.^17^ propose a method called TL with false negative control (FNC), which combines FNC marginal screening in the base cohort with joint model training to improve PRS prediction performance in the target cohort. It demonstrates the potential of TL to enhance PRS prediction accuracy and reduce bias across diverse populations. In addition, another method called TL-Multi has been recently developed to leverage summary statistics from multiple ancestral populations to improve PRS prediction in target populations^16^. It uses a high-dimensional linear regression model to effectively transfer knowledge across different populations. All these examples show that TL holds great promise for improving PRS prediction accuracy in target populations with limited sample sizes and/or data heterogeneity. However, such TL-based methods or frameworks have not been explored in the drug response and PGx space for cross-phenotype (disease to drug response) prediction, where sample sizes in target PGx cohorts are usually small and the genetic architecture of a drug response usually differs from that of the related disease (i.e., not all disease-associated SNPs may be relevant for drug response prediction). This motivates us to explore the TL strategy in the case of domain shift (i.e., from disease domain to drug response domain) and develop more powerful statistical methods in terms of their capability of capturing the relationship between the disease-associated SNPs and drug response.

In this paper, we propose a novel transfer learning (TL) based method called PRS-PGx-TL, which constructs PRS for drug response prediction and patient stratification by jointly modeling disease GWAS data (e.g., disease GWAS summary statistics in base cohort) and PGx GWAS data (e.g., individual-level PGx data in target cohort), and simultaneously estimating both prognostic and predictive effects. In PRS-PGx-TL, we further develop a two-dimensional penalized gradient descent algorithm, which takes the SNP weights from the disease GWAS as initial values and optimizes the tuning parameters using a cross-validation framework to update both prognostic and predictive effects. Gradient descent is an optimization algorithm aiming to find the minimum of a function through iteratively adjusting parameters in the opposite direction of the function’s gradient or slope. The optimization problem in PRS-PGx-TL involves two variables: the main genetic (G) effect (i.e., prognostic effect) and the genotype by treatment interaction (GTI) effect (i.e., predictive effect). More specifically, traditional disease PRS methods are used on the large-scale disease GWAS summary statistics data to estimate the initial weights for the SNPs. Then these weights are used as the starting point of the prognostic (or G) effect for the target PGx GWAS data. The proposed two-dimensional penalized gradient descent algorithm is used to fine-tune the model on the target data with both prognostic (or G) effect and predictive (or GTI) effect. This allows the model to transfer the knowledge from disease GWAS to PGx GWAS and adapt to the specific characteristics of the PGx population and drug response. In addition, compared with the gradient descent algorithm without a penalized term, our proposed penalized gradient descent algorithm encourages the algorithm to find solutions with sparsity and prevents overfitting. Given the base disease GWAS data does not provide any predictive (or GTI) effects, we employ two strategies to initialize the starting values of the predictive effects: either starting from zero or from the same initial prognostic effects estimated from the base cohort. Likewise, we adopt two approaches to update the effects: updating either both prognostic and predictive effects or the predictive effect only (by assuming the prognostic effect is unchanged). Additionally, we introduce two criteria for parameter tuning: maximizing either overall *R*^2^, including both G and GTI effects (i.e., focusing on the optimization of both prognostic and predictive effects) or *R*^2^ of GTI conditional on G (i.e., focusing more on the optimization of predictive effects) in the two-dimensional penalized gradient descent algorithm. In total, we implement six different strategies (or sub-methods M1–M6) in the PRS-PGx-TL framework.

Our extensive simulations show that PRS-PGx-TL methods generally improve prediction accuracy and population stratification performance compared to the traditional disease PRS methods (e.g., PRS-CS, Lassosum) although the performance of the six models of PRS-PGx-TL varies. The improvements are robust across a variety of simulation scenarios. In the application to IMPROVE-IT PGx GWAS data, we further demonstrate that PRS-PGx-TL methods achieve higher drug response prediction accuracy and better patient stratification performance while predicting treatment-related LDL cholesterol reduction. By incorporating genotype by treatment interactions into the two-dimensional penalized gradient descent framework and solving the 2-dimensional PGx PRS optimization problem, PRS-PGx-TL models are shown to help improve drug response prediction and facilitate precision medicine.

## Results

### Workflow of PRS-PGx-TL

**Fig. 1:**
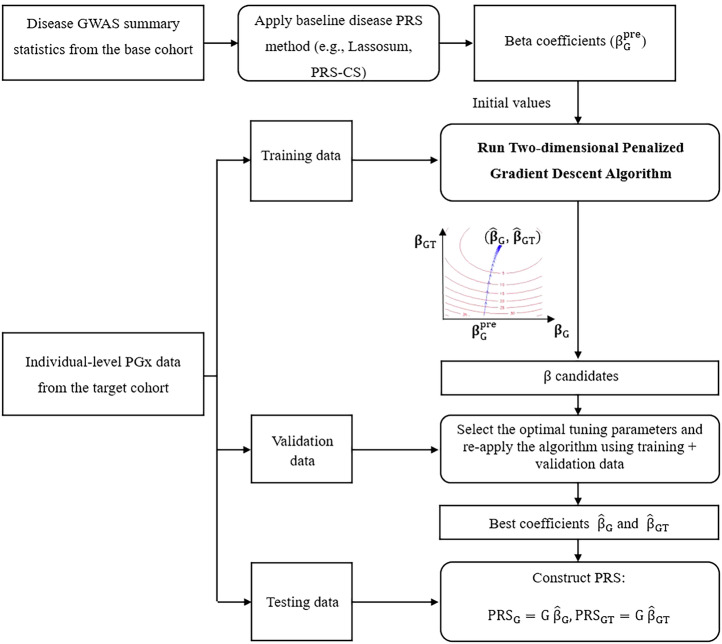
PRS-PGx-TL workflow. Disease GWAS summary statistics in the base cohort are first used to calculate beta coefficients (${\beta }_{{\rm{G}}}^{\mathrm{pre}}$) as initial values via baseline disease PRS methods. Individual-level PGx data in the target cohort is split into training, validation, and testing datasets via the nested cross-validation procedure. In each repeat, the training dataset, together with the initial values, is used to train the two-dimensional penalized gradient model; the validation dataset is used to select the optimal tuning parameters; prognostic and predictive PRSs are constructed in the testing dataset. The graph was created with the open-source animation R package (https://cran.r-project.org/package=animation).

We first built (or trained) models using existing disease PRS methods, and the effect estimates of those pre-trained baseline models (Lassosum, PRS-CS) were used as initial values for PRS-PGx-TL. Given the lack of availability of an independent PGx GWAS validation dataset, nested cross-validation was used to apply PRS-PGx-TL to the individual-level PGx GWAS data (in the target cohort). The individual-level PGx data was first split into five folds in the “outer layer” of cross-validation with four allocated for training and the rest one for testing. The two-dimensional penalized gradient descent algorithm (details in section “Methods”) was then applied to each of the training sets. More specifically, the SNP weights from the pre-trained disease model were taken as the initial values, and the training set was further split into four folds (“inner layer” of cross-validation), three for training and one for validation, to select the optimal tuning parameters (learning rate $\eta$, regularization parameter $\lambda$, and number of iterations *r*). Then the two-dimensional penalized gradient descent algorithm was re-applied with the optimal parameters on the full training data (training + validation) to get the final weights of PRS-PGx-TL: prognostic effects ${\hat{{\boldsymbol{\beta }}}}_{{\rm{G}}}$ and predictive effects ${\hat{{\boldsymbol{\beta }}}}_{{\rm{GT}}}$. Given the weights from the algorithm, we constructed the two final PRSs from the testing set: ${\rm{PR}}{{\rm{S}}}_{{\rm{G}}}={\rm{G}}{\hat{{\boldsymbol{\beta }}}}_{{\rm{G}}}$ and ${\rm{PR}}{{\rm{S}}}_{{\rm{GT}}}={\rm{G}}{\hat{{\boldsymbol{\beta }}}}_{{\rm{GT}}}$ (Fig. 1). By iterating the process through all five folds of the “outer layer”, we could obtain the PRS for all the individuals in the PGx data (the target cohort), including both prognostic and predictive PRSs. Of note, by using different implementation strategies, we designed a total of six models of PRS-PGx-TL depending on: (1) whether to update ${{\boldsymbol{\beta }}}_{{\bf{G}}}$ and ${{\boldsymbol{\beta }}}_{{\bf{GT}}}$ at the same time, or fix ${{\boldsymbol{\beta }}}_{{\bf{G}}}={{\boldsymbol{\beta }}}_{{\rm{G}}}^{{\rm{pre}}}$ and only update ${{\boldsymbol{\beta }}}_{{\bf{GT}}}$, (2) whether to set the initial values for ${{\boldsymbol{\beta }}}_{{\bf{GT}}}$ to 0 or ${{\boldsymbol{\beta }}}_{{\rm{G}}}^{{\rm{pre}}}$, (3) whether to maximize the overall *R*^2^ or the conditional ${\rm{PR}}{{\rm{S}}}_{{\rm{GT}}}\times {\rm{T}}$ *R*^2^ (conditional on ${\rm{PR}}{{\rm{S}}}_{{\rm{G}}}$) as a criterion for parameter tuning. The details of the six models are summarized in Table 1.

**Table 1:** PRS-PGx-TL: model summary with six different implementation strategies

Model	Update ${{\rm{\beta }}}_{{\rm{G}}}$ or ${{\rm{\beta }}}_{{\rm{GT}}}$	Initial values	Criterion for parameter tuning
M1 (${{\rm{\beta }}}_{{\rm{G}}}$&${{\rm{\beta }}}_{{\rm{GT}}}$, 0-init and G&GTI-focused)	Update both	${{\rm{\beta }}}_{{\rm{G}}}={{\rm{\beta }}}_{{\rm{G}}}^{{\rm{pre}}}$ ${{\rm{\beta }}}_{{\rm{GT}}}=0$	Maximize overall *R*^2^
M2 (${{\rm{\beta }}}_{{\rm{G}}}$&${{\rm{\beta }}}_{{\rm{GT}}}$, ${{\rm{\beta }}}_{{\rm{G}}}^{{\rm{pre}}}$-init and G&GTI-focused)	Update both	${{\rm{\beta }}}_{{\rm{G}}}={{\rm{\beta }}}_{{\rm{G}}}^{{\rm{pre}}}$ ${{\rm{\beta }}}_{{\rm{GT}}}={{\rm{\beta }}}_{{\rm{G}}}^{{\rm{pre}}}$	Maximize overall *R*^2^
M3 (${{\rm{\beta }}}_{{\rm{G}}}$&${{\rm{\beta }}}_{{\rm{GT}}}$, 0-init and GTI-focused)	Update both	${{\rm{\beta }}}_{{\rm{G}}}={{\rm{\beta }}}_{{\rm{G}}}^{{\rm{pre}}}$ ${{\rm{\beta }}}_{{\rm{GT}}}=0$	Maximize conditional *R*^2^ of ${\rm{PR}}{{\rm{S}}}_{{\rm{GT}}}\times {\rm{T}}$ (conditional on ${\rm{PR}}{{\rm{S}}}_{{\rm{G}}}$)
M4 (${{\rm{\beta }}}_{{\rm{G}}}$&${{\rm{\beta }}}_{{\rm{GT}}}$, ${{\rm{\beta }}}_{{\rm{G}}}^{{\rm{pre}}}$-init and GTI-focused)	Update both	${{\rm{\beta }}}_{{\rm{G}}}={{\rm{\beta }}}_{{\rm{G}}}^{{\rm{pre}}}$ ${{\rm{\beta }}}_{{\rm{GT}}}={{\rm{\beta }}}_{{\rm{G}}}^{{\rm{pre}}}$	Maximize conditional *R*^2^ of ${\rm{PR}}{{\rm{S}}}_{{\rm{GT}}}\times {\rm{T}}$ (conditional on ${\rm{PR}}{{\rm{S}}}_{{\rm{G}}}$)
M5 (${{\rm{\beta }}}_{{\rm{GT}}}$, 0-init and G&GTI-focused)	Only update ${{\rm{\beta }}}_{{\rm{GT}}}$(fix ${{\rm{\beta }}}_{{\rm{G}}}={{\rm{\beta }}}_{{\rm{G}}}^{{\rm{pre}}}$)	${{\rm{\beta }}}_{{\rm{GT}}}=0$	Maximize overall *R*^2^ (equivalent to maximize conditional *R*^2^)
M6 (${{\rm{\beta }}}_{{\rm{GT}}}$, ${{\rm{\beta }}}_{{\rm{G}}}^{{\rm{pre}}}$-init and G&GTI-focused)	Only update ${{\rm{\beta }}}_{{\rm{GT}}}$(fix ${{\rm{\beta }}}_{{\rm{G}}}={{\rm{\beta }}}_{{\rm{G}}}^{{\rm{pre}}}$)	${{\rm{\beta }}}_{{\rm{GT}}}={{\rm{\beta }}}_{{\rm{G}}}^{{\rm{pre}}}$	Maximize overall *R*^2^ (equivalent to maximize conditional *R*^2^)

These six models differ from (1) how to update ${{\boldsymbol{\beta }}}_{{\bf{G}}}$ and/or ${{\boldsymbol{\beta }}}_{{\bf{GT}}}$ (${{\rm{\beta }}}_{{\rm{G}}}$&${{\rm{\beta }}}_{{\rm{GT}}}$: update both, ${{\rm{\beta }}}_{{\rm{GT}}}$: update only ${{\rm{\beta }}}_{{\rm{GT}}}$ by fixing ${{\rm{\beta }}}_{{\rm{G}}}$), (2) how to set initial values (0-init: ${{\rm{\beta }}}_{{\rm{GT}}}=0,\,{{\rm{\beta }}}_{{\rm{G}}}^{{\rm{pre}}}$-init: ${{\rm{\beta }}}_{{\rm{GT}}}={{\rm{\beta }}}_{{\rm{G}}}^{{\rm{pre}}}$), and (3) how to set the criterion for parameter tunning (G&GTI-focused: maximize overall *R*^2^, GTI-focused: maximize *R*^2^ of ${\rm{PR}}{{\rm{S}}}_{{\rm{GT}}}\times {\rm{T}}$ conditional on ${\rm{PR}}{{\rm{S}}}_{{\rm{G}}}$).

### Simulation results

In the simulations, we mainly contrasted PRS-PGx-TL with two disease PRS methods: Lassosum and PRS-CS across different setting of ${\rho }_{\mathrm{DT}}$ (effect correlation between the trait in the base cohort and the drug response in the target cohort) and ${\rho }_{{\rm{E}}}$ (correlation between prognostic and predictive effects in PGx data). More details of the parameter setup are provided in Table S1. The performance was assessed in an independent testing set (*n* = 1000) in terms of (1) overall prediction accuracy ${R}^{2}$ between the observed and predicted phenotypes, (2) the predictive *p* value of ${\rm{PR}}{{\rm{S}}}_{{\rm{GT}}}$-by-treatment interaction (on −log10 scale), (3) partial ${R}^{2}$ explained by the ${\rm{PR}}{{\rm{S}}}_{{\rm{G}}}$ term, (4) partial ${R}^{2}$ explained by the ${\rm{PR}}{{\rm{S}}}_{{\rm{GT}}}\times {\rm{T}}$ term (conditional on ${\rm{PR}}{{\rm{S}}}_{{\rm{G}}}$). More details are provided in section “Methods”.

**Fig. 2:**
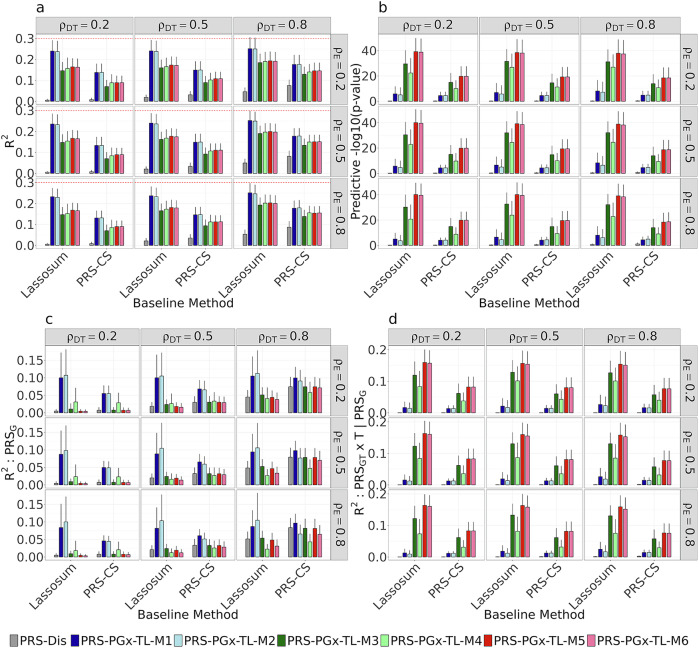
Simulation performance comparisons between disease PRS methods (Lassosum and PRS-CS) and PRS-PGx-TL method with six different implementation strategies (M1–M6). Methods are compared in terms of **a** overall ${{\bf{R}}}^{{\bf{2}}}$, **b** predictive *p* value, **c** partial ${{\bf{R}}}^{{\bf{2}}}$ explained by the ${\bf{PR}}{{\bf{S}}}_{{\bf{G}}}$ term, and **d** partial ${{\bf{R}}}^{{\bf{2}}}$ explained by the ${\bf{PR}}{{\bf{S}}}_{{\bf{GT}}}{\boldsymbol{\times }}{\bf{T}}$ term (conditional on ${\bf{PR}}{{\bf{S}}}_{{\bf{G}}}$). ${{\bf{H}}}_{{\bf{D}}}^{{\bf{2}}}=0.3$, ${\boldsymbol{\gamma }}=1$, **pcausal** = 0.01, ${{\boldsymbol{\rho }}}_{{\bf{DT}}}\in \{0.2,\,0.5,\,0.8\}$, ${{\boldsymbol{\rho }}}_{{\bf{E}}}\in \{0.2,\,0.5,\,0.8\}$. The graph was created with the open-source ggplot2 R package (https://ggplot2.tidyverse.org).

The prediction performance of the PRS-Dis method, and six models of PRS-PGx-TL corresponding to each of the two baseline methods (Lassosum and PRS-CS) is summarized in Fig. 2. The PRS-PGx-TL methods generally outperformed the corresponding PRS-Dis methods. For example, PRS-PGx-TL-M1 to M6 all improved the overall prediction accuracy ${R}^{2}$ compared to the PRS-Dis method, with M1 and M2 achieving the highest ${R}^{2}$ among the six models (Fig. 2a). For the predictive *p* value (Fig. 2b), all six TL-based models showed improved power to detect the predictive effect by increasing the −log_10_($\mathrm{PR}{{\rm{S}}}_{\mathrm{GT}}\times T$ interaction *p* value), with models M3–M6 having the most substantial improvements. In contrast, M1 and M2 had the greatest increase in partial ${R}^{2}$ for the ${\rm{PR}}{{\rm{S}}}_{{\rm{G}}}$ term (Fig. 2c), indicating their improvement in the overall ${R}^{2}$ was mainly contributed by the disease genetic effect G. The partial ${R}^{2}$ for $\mathrm{PR}{{\rm{S}}}_{\mathrm{GT}}\times T$ term showed a similar pattern as the predictive *p* value, where M3–M6 generally performed better (Fig. 2d). We noted that M3–M6 all put more emphasis on the predictive effects by either maximizing the conditional *R*^2^ of $\mathrm{PR}{{\rm{S}}}_{\mathrm{GT}}\times T$ in parameter tuning (in M3, M4) or only updating ${{\boldsymbol{\beta }}}_{{\bf{GT}}}$ while keeping ${{\boldsymbol{\beta }}}_{{\bf{G}}}$ fixed (in M5, M6), which explains their superior performance in terms of predictive p-value and the partial ${R}^{2}$ for $\mathrm{PR}{{\rm{S}}}_{\mathrm{GT}}\times T$ term. We also contrasted the patient stratification performances of different methods by plotting the average treatment effect versus different PRS quantiles (Fig. S1). PRS-PGx-TL-M1 to M6 showed a much clearer increasing trend than the PRS-Dis methods, indicating the PRS-PGx-TL methods had better performance in patient stratification.

We observe that the performance of all methods increased with ${\rho }_{\mathrm{DT}}$, especially for the PRS-Dis methods. For example, the overall ${R}^{2}$ for the PRS-Dis methods increased from slightly above 0 to 0.1 when ${\rho }_{\mathrm{DT}}$ increased from 0.2 to 0.8 (Fig. 2a). This was as expected since ${\rho }_{\mathrm{DT}}$ reflects the similarity between the disease GWAS in the base cohort and the drug response in the PGx target cohort. When the two different datasets are more similar (higher ${\rho }_{\mathrm{DT}}$), the PRS-Dis methods which only utilize the disease GWAS could have better performance in predicting the drug response in the PGx cohort. In this case, the additional increased performance of PRS-PGx-TL methods may also link to the fact that the base (disease) cohort is typically larger with more substantial data; being able to borrow more strength from this (or use only this) can vastly improve prediction performance. There was no obvious change in the performance of any of the evaluated PRS-PGx-TL methods with the increase of ${\rho }_{{\rm{E}}}$, which measured the correlation between prognostic and predictive effects in PGx GWAS. This indicates that the prediction accuracy and the power to detect the predictive effect of our proposed models were generally robust under different correlations between prognostic and predictive effects. While comparing the two baseline methods, we found that the PRS-PGx-TL models using Lassosum generally had superior performances than those using PRS-CS. This suggests that the TL framework is generally better suited for sparse models like Lassosum, which reduces the number of noisy SNPs likely to enter the gradient descent step.

We also included C + T as another baseline disease PRS method in the simulations. Since it does not have an auto-version and requires independent validation data to tune parameters, we summarized its simulation results separately from Lassosum and PRS-CS (auto-versions without validation data). Similar patterns were observed for the predictive *p* value and the partial ${R}^{2}$ for $\mathrm{PR}{{\rm{S}}}_{\mathrm{GT}}\times T$ term (Fig. S2). Although PRS-PGx-TL utilizes both disease summary statistics and individual-level PGx data for drug response prediction, the individual-level PGx data also provides an opportunity to directly train a PRS-PGx model without relying on transfer learning. While it utilized a cross-validation framework rather than independent training data, its performance is still worth investigating as another baseline comparator. Therefore, we conducted an additional simulation analysis using only individual-level PGx data to train a direct PRS-PGx, which was under the same cross-validation framework as PRS-PGx-TL, but the initial values ${{\boldsymbol{\beta }}}_{{\bf{G}}}$ and ${{\boldsymbol{\beta }}}_{{\bf{GT}}}$ were set to 0 so that information from the disease (base) cohort was not “transferred”. In this case, the results from only PRS-PGx-M1 and PRS-PGx-M3 were reported along with those from other methods. The results (Fig. S3) showed that the PRS-PGx method had a better performance than the PRS-Dis method, but PRS-PGx-TL still achieved higher ${R}^{2}$ than the baseline PRS-PGx or PRS-Dis methods. For example, PRS-PGx-TL-M1 and M2 had the highest overall prediction accuracy ${R}^{2}$ among all models (Fig. S3a), and M3–M6 showed the greatest improvements in −log_10_($\mathrm{PR}{{\rm{S}}}_{\mathrm{GT}}\times T$ interaction *p* value) (Fig. S3b).

### Simulation results: sensitivity analyses

**Fig. 3:**
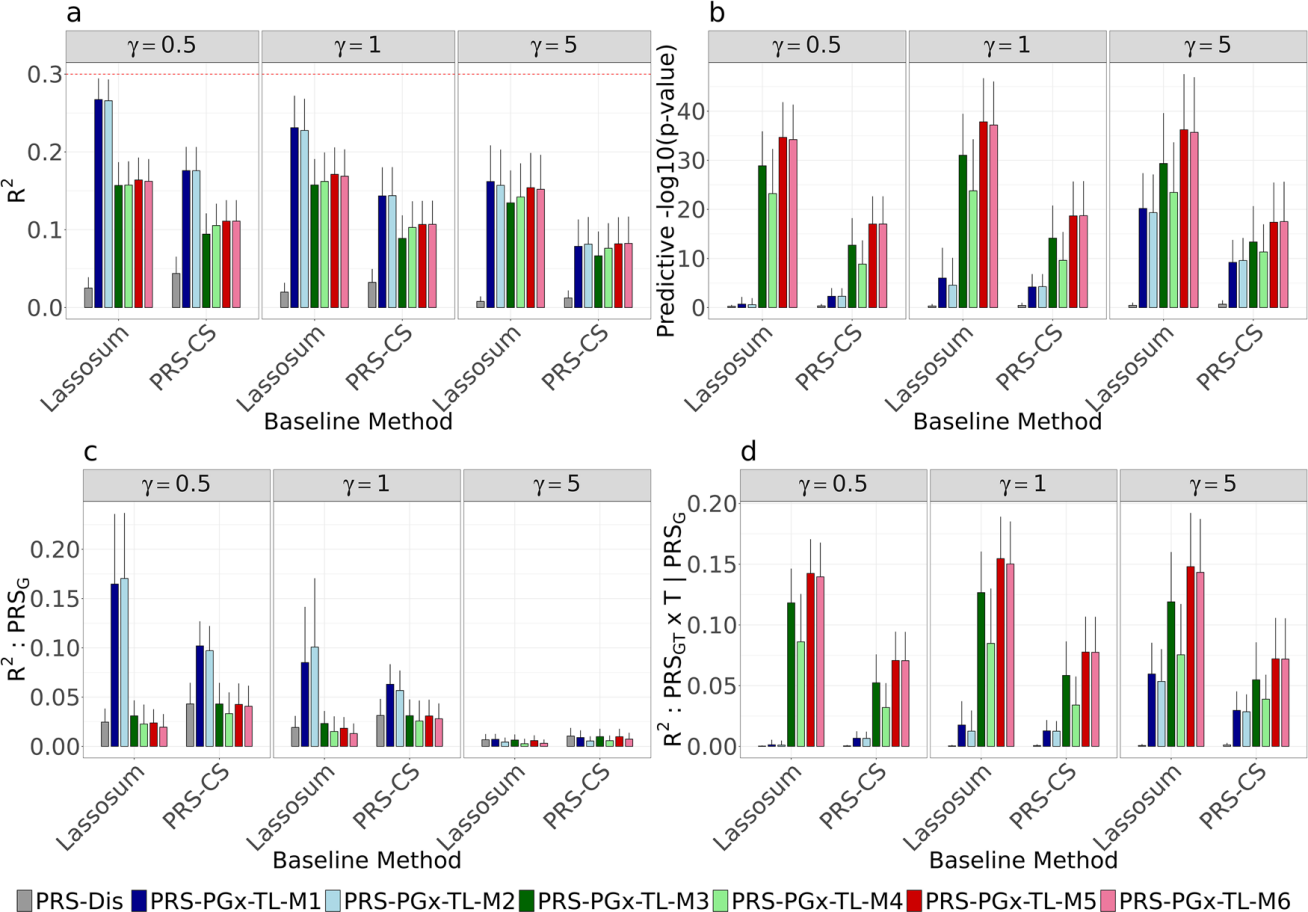
Simulation performance comparisons between disease PRS methods (Lassosum and PRS-CS) and PRS-PGx-TL method with six different implementation strategies (M1–M6). Methods are compared in terms of **a** overall ${{\bf{R}}}^{{\bf{2}}}$, **b** predictive *p* value, **c** partial ${{\bf{R}}}^{{\bf{2}}}$ explained by the ${\bf{PR}}{{\bf{S}}}_{{\bf{G}}}$ term, and **d** partial ${{\bf{R}}}^{{\bf{2}}}$ explained by the ${\bf{PR}}{{\bf{S}}}_{{\bf{GT}}}{\boldsymbol{\times }}{\bf{T}}$ term (conditional on ${\bf{PR}}{{\bf{S}}}_{{\bf{G}}}$). ${{\bf{H}}}_{{\bf{D}}}^{{\bf{2}}}={\bf{0}}{\boldsymbol{.}}{\bf{3}}$, ${\boldsymbol{\gamma }}{\boldsymbol{\in }}{\boldsymbol{\{}}{\bf{0}}{\boldsymbol{.}}{\bf{5}},\,{\bf{1}},\,{\bf{5}}{\boldsymbol{\}}}$, **pcausal** = **0.01,** ${{\boldsymbol{\rho }}}_{{\bf{DT}}}={\bf{0}}{\boldsymbol{.}}{\bf{5}}$, ${{\boldsymbol{\rho }}}_{{\bf{E}}}={\bf{0}}{\boldsymbol{.}}{\bf{5}}$. The graph was created with the open-source ggplot2 R package (https://ggplot2.tidyverse.org).

To further assess the performance of the proposed methods, we conducted additional simulations varying key parameters: $\gamma$ (scaling factor of predictive effect to prognostic effect), pcausal (proportion of causal SNPs), ${h}_{{\rm{D}}}^{2}$ (heritability to explain the drug response in the target cohort), and proportions of joint causal variants on both the disease and the drug response. The predictive performance of the PRS-Dis methods, and the corresponding six models of PRS-PGx-TL under different scales of the prognostic and predictive effect sizes are summarized in Fig. 3. We increased $\gamma$ from 0.5 to 5 to let the predictive effects become more dominant (i.e., more heritability is explained by the predictive effects when $\gamma =5$). Along this process, PRS-PGx-TL methods generally outperformed the corresponding PRS-Dis methods, though all methods showed a decrease in overall ${R}^{2}$ (Fig. 3a) and an increase in predictive *p* value (in −log10 scale) (Fig. 3b). We also found that M1 and M2 had bigger changes than M3–M6. For example, using Lassosum as the baseline model, the partial ${R}^{2}$ for PRS_G_ term decreased dramatically from 0.165 to 0.007 for M1 and from 0.17 to 0.004 for M2 (Fig. 3c). In addition, the partial ${R}^{2}$ for $\mathrm{PR}{{\rm{S}}}_{\mathrm{GT}}\times T$ interaction term increased substantially from 0.001 to 0.06 for M1 and from 0.001 to 0.053 for M2 (Fig. 3d). The different implementation strategies of these models resulted in different result patterns: M3–M6 put more emphasis on the predictive effects and M1–M2 focused more emphasis on the overall ${R}^{2}$. Therefore, M3–M6 could maintain a lower predictive *p* value (higher in −log10 scale) and higher partial ${R}^{2}$ for the $\mathrm{PR}{{\rm{S}}}_{\mathrm{GT}}\times T$ term no matter how the scales of predictive effect to prognostic effect changed. In contrast, M1–M2 were more sensitive to this process.

We also conducted sensitivity analyses by varying the proportion of causal SNPs, the heritability, and the proportions of joint causal variants on both the disease and the drug response. When we increased the proportion of causal SNPs (i.e., moving from a sparse setting to a polygenic setting), the performance decreased for all Lassosum-based models and did not change obviously for the PRS-CS-based model (Fig. S4). For example, with the increase in the proportion of causal SNPs, all Lassosum-based models had lower power to detect the predictive effect, reflected by the decreased interaction *p* value in −log10 scale. In contrast, the performance of PRS-CS-based models did not have a decreasing pattern, and PRS-PGx-TL-M5 and M6 even had slightly increased power. This was not surprising since Lassosum penalizes the effect size of many SNPs to zero and filters those SNPs out. When the setting becomes more polygenic, this might lead to the loss of informative SNPs and result in decreased performance. PRS-CS, however, uses a Bayesian framework and does not shrink SNP effect sizes to exact zero, keeping all SNPs in the gradient descent algorithm and thus leading to more robust performances in polygenic settings. When we decreased the heritability (i.e., to ${h}_{{\rm{D}}}^{2}=0.1$), all methods had worse performances (Fig. S5). However, PRS-PGx-TL methods continued to demonstrate improved performance compared to the corresponding PRS-Dis methods across all the settings. We explored a more complex scenario by varying the proportions of joint causal variants, meaning not all causal variants affected both disease and drug response; instead, some were disease-specific, and others were drug-response-specific. We conducted an additional simulation analysis under two additional scenarios—scenario A: 80% of causal variants affected both disease and drug response, while 10% exclusively affected disease and 10% exclusively affected drug response; and scenario B: 60% of causal variants affected both disease and drug response, with 20% specific to disease and 20% specific to drug response. We compared these results to our original scenario, where 100% of causal variants were assumed to affect both disease and drug response. The simulation results were summarized in Fig. S6. As the proportion of joint causal variants decreased, all methods saw a slight drop in performance (Fig. S6). However, the PRS-PGx-TL methods consistently maintained higher ${R}^{2}$ values than the corresponding PRS-Dis methods, demonstrating their robust performance even when different sets of causal variants influence disease and drug response.

### IMPROVE-IT PGx GWAS data analysis results

We applied two disease PRS methods (Lassosum and PRS-CS) and their corresponding PRS-PGx-TL methods (M1, M3, M5) to the IMPROVE-IT PGx GWAS data to predict the LDL-C log-fold change at 1-month. PRS-PGx-TL M2, M4, M6 (with ${{\rm{\beta }}}_{\mathrm{GT}}={{\rm{\beta }}}_{{\rm{G}}}^{\mathrm{pre}}$ as a start point) were not included as our simulations clearly showed that M1, M3, M5 (with ${{\rm{\beta }}}_{\mathrm{GT}}=0$ as a start point) generally outperformed them, respectively.

The prediction performance was measured by overall prediction accuracy ${R}^{2}$, conditional ${R}^{2}$ and ${\rm{PR}}{{\rm{S}}}_{{\rm{GT}}}$-by-treatment interaction *p* value, with the results summarized in Table 2. Consistent with the simulation results, the PRS-PGx-TL methods had an overall improvement in both ${R}^{2}$ and predictive *p* value. For example, PRS-PGx-TL-M1 increased the overall prediction accuracy ${R}^{2}$ from 0.048 to 0.051, and from 0.072 to 0.075 when using Lassosum and PRS-CS as the baseline methods, respectively. The improvement in conditional ${R}^{2}$ was marginal, but a more obvious improvement in predictive *p* value was observed. For instance, the predictive *p* values using the disease PRS methods were 0.388 (Lassosum) and 0.063 (PRS-CS); both were not significant. After applying PRS-PGx-TL, M5 achieved highly significant predictive *p* values: 2.83e−06 for Lassosum and 9.60e−05 for PRS-CS.

**Table 2:** Method comparisons between disease PRS methods (Lassosum, and PRS-CS) and PRS-PGx-TL method with different implementation strategies (M1, M3, M5) from their applications to the IMPROVE-IT PGx GWAS data

PRS method	# of SNPs	*R*^2^	*R*^2^ for ${\rm{PR}}{{\rm{S}}}_{{\rm{G}}}$	*R*^2^ for ${\rm{PR}}{{\rm{S}}}_{{\rm{GT}}}\times {\rm{T\; |\; PR}}{{\rm{S}}}_{{\rm{G}}}$	Predictive *p* value	${{\rm{\beta }}}_{{\rm{GT}}}$
PRS-Dis (Lassosum)	30,756	0.048	0.048	9.14e−05	0.388	−0.018
PRS-PGx-TL-M1	30,756	0.051	0.048	0.001	0.0045	0.017
PRS-PGx-TL-M3	30,756	0.051	0.048	0.003	1.12e−04	0.024
PRS-PGx-TL-M5	30,756	0.052	0.048	0.004	2.83e−06	0.028
PRS-Dis (PRS-CS)	1,109,919	0.072	0.071	3.58e−04	0.063	−0.044
PRS-PGx-TL-M1	1,109,919	0.075	0.073	0.002	0.0036	0.017
PRS-PGx-TL-M3	1,109,919	0.070	0.066	2.62e−04	0.188	0.008
PRS-PGx-TL-M5	1,109,919	0.074	0.072	0.003	9.60e−05	0.024

**Fig. 4:**
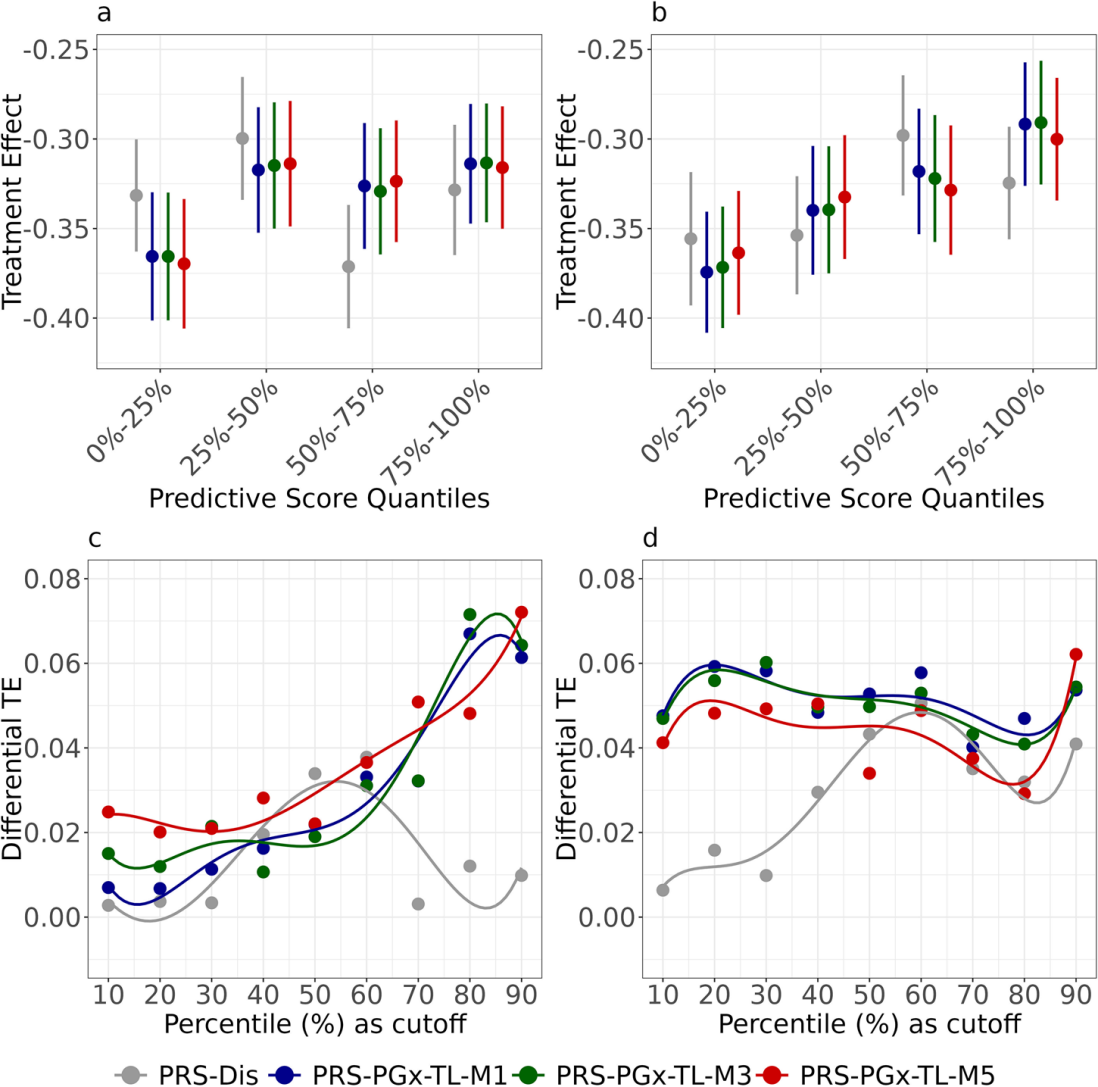
Patient stratification results from the PRS analysis of the IMPROVE-IT PGx GWAS data. Quantile plots of treatment effect using four fixed quantiles (0–25%, 25–50%, 50–75%, and 75–100%) with different baseline methods: **a** Lassosum, **b** PRS-CS. Each dot stands for the observed Treatment Effect (TE), and each bar denotes the 95% Confidence Interval (CI). Differential treatment effect when patients were stratified into top 10%, 20%, ⋯ , 90% percentile of the predictive score vs. the rest, respectively, with different baseline methods: **c** Lassosum, **d** PRS-CS. The graph was created with the open-source ggplot2 R package (https://ggplot2.tidyverse.org).

We further compared the patient stratification performance across different methods and summarized the results in Fig. 4. In Fig. 4a, b, the treatment effect differences between treatment and control arms versus four fixed quantiles (0–25%, 25–50%, 50–75%, and 75–100%) were compared across the methods. A clear increasing or decreasing pattern indicated that the treatment effects varied consistently across different patient subgroups stratified by the predictive score. The predictive score determined by PRS-PGx-TL methods (M1, M3, M5) showed a much clearer increasing pattern than their corresponding disease PRS methods, demonstrating the superiority of PRS-PGx-TL for patient stratification. In Fig. 4c, d, patients were stratified into two groups using nine different cutoffs: the top 10%, 20%, ⋯, 90% percentile of the predictive score. The corresponding difference in treatment effect between the two groups was calculated and plotted versus the cutoffs used. We observed that the differential treatment effects of the PRS-PGx-TL methods (M1, M3, M5) were generally larger than the disease PRS methods across different cutoff points. Altogether, PRS-PGx-TL models yielded a more meaningful performance in patient stratification compared to the disease PRS methods.

The distributions of the prognostic and predictive effect sizes of the whole genome SNPs estimated by PRS-PGx-TL methods (M1, M3, M5) are displayed in Fig. S7 (Lassosum used as the baseline method) and Fig. S8 (PRS-CS used as the baseline method). Both figures show that most of the models identified the top signals on chromosome 19. The corresponding annotation information of the top 20 SNPs with the largest predictive effect sizes estimated by PRS-PGx-TL is summarized in Tables S2 and S3. Some of the signals, including rs7254892 (mapped to *PVRL2*) and rs7412 (mapped to *APOE*), are supported by the previous association evidence from literature^18,19^. We also contrasted the PRS estimated by PRS-PGx-TL using two different baseline methods (Lassosum vs. PRS-CS) and summarized the results in Fig. S9. The prognostic PRS values between Lassosum-based and PRS-CS-based models had a higher correlation than the predictive PRS. For example, in M1 the Pearson’s correlations for the prognostic effects and predictive effects were 0.89 and 0.39, respectively. This pattern was consistent across M1, M3 and M5.

### Computation time

To assess the computational burden of the proposed method, we summarized the computation time of PRS-PGx-TL in both simulations and real data analysis in (Table S4). In simulations, we included a total of 5000 individuals and 20,854 SNPs on chromosome 19, and the average and standard deviation (SD) of the computational time across 1000 repeats were reported. Using Lassosum as the baseline method, M1–M4 had similar computation times at around 3 min, while M5-M6 had a faster speed at around 2 min. This was as expected since M5–M6 only updated ${{\boldsymbol{\beta }}}_{{\bf{GT}}}$ with ${{\boldsymbol{\beta }}}_{{\bf{G}}}$ fixed. When PRS-CS was used as the baseline method, the computation time was longer: M1–M4 at around 17 min and M5–M6 at around 9 min. In real (IMPROVE-IT GWAS) data analysis, a total of 5661 subjects and 1,109,919 SNPs were included, and parallel computing was used to compute the five folds of the “outer layer” and 22 chromosomes simultaneously. Similarly, PRS-PGx-TL based on Lassosum (M1–M4 at around 6 min, M5–M6 at around 4 min) computed faster than that based on PRS-CS (M1–M4 at around 63 min, M5–M6 at around 34 min).

## Discussion

The main contributions of this paper are two-fold. First, we creatively apply the Transfer Learning based framework to PRS modeling in the PGx space. In this framework, information from a disease GWAS is leveraged to predict drug response in a PGx GWAS. In other words, transfer learning provides great value in adapting an existing PRS model in a disease population to a new PGx population, which is essentially a case for domain shift (i.e., from disease domain to drug response domain) based PRS prediction. This TL-based framework enables us to understand the shared genetic architectures between disease and drug response and links two types of knowledge together in PRS modeling. Second, we propose a novel two-dimensional penalized gradient descent algorithm to fine-tune the model parameters and transfer the knowledge from disease domain to drug response domain. By simultaneously modeling the prognostic and predictive effects and incorporating regularization (or a penalty term) in the optimization problem, PRS-PGx-TL can enhance model performance, improve generalizability, and facilitate the development of more accurate and robust PRS models for PGx applications.

Despite both relying on transfer learning techniques, our PRS-PGx-TL approach differs from traditional cross-ancestry PRS methods in two primary ways: (1) our approach focuses on phenotype transfer (from disease to drug response), which is more like a heterogeneous (or domain shift) transfer learning problem, while the cross-ancestry PRS methods are more like a homogeneous transfer learning problem. Within a homogeneous transfer learning problem, the feature spaces of the data in the source and target domains are of the same dimension and the transfer learning problem focuses on bridging the gap in the data distributions between the domains. But in our case or within a heterogeneous transfer learning problem, the data are heterogeneous—disease and drug response phenotypes are different, leading to additional domain shift issues. Furthermore, the features are also heterogeneous; for instance, not all disease-associated SNPs are relevant for drug response prediction, and vice versa. (2) Our approach transfers knowledge from disease to drug response in two-dimensional space (genotype main effect and genotype-by-treatment interaction effect or prognostic and predictive effects) while the traditional cross-ancestry PRS methods transfer knowledge from one large population (i.e., European) to another small or underrepresented population (i.e., African) in one-dimensional space (i.e., focusing on only genotype effect). Compared with traditional transfer learning cross-ancestry PRS methods, our proposed method is designed for handling more complex transfer learning problems, particularly in the individualized fine-tuning of genotype-by-treatment interaction or predictive effects, which is a key innovation that sets it apart from PRS-Dis and direct PGx PRS approaches.

Genetic susceptibility to a specific disease (or multiple related diseases) may predispose to a drug response. However, the strength of this link often remains a major unanswered question in most PRS applications within PGx studies. In addition, developing PRS requires significant patient numbers, which is a general issue in the PGx space where the sample size is usually small. How accurate does a PRS built from a genetically related disease, which is much more common (i.e., with much larger sample size), predict a drug response? Our proposed PRS-PGx-TL framework and methods help address such questions.

We implement the algorithm in a flexible way with six potential strategies (M1–M6), with different optimization goals and different starting values of predictive effect, allowing different choices to be made according to specific research interests. Our extensive simulation studies show that PRS-PGx-TL (M1–M6) outperforms the PRS-Dis method; M1 and M2 have substantial improvement in overall *R*^2^ and *R*^2^ for prognostic (G) effect and M3–M6 primarily decrease the predictive *p* value and increase the conditional *R*^2^ for predictive (GTI) effect. In addition, PRS-PGx-TL (M1–M6) yields better stratification performance than the PRS-Dis method. Furthermore, M1, M3, and M5 (by assuming the initial value of ${{\rm{\beta }}}_{\mathrm{GT}}$ = 0) generally outperform M2, M4, and M6 (by assuming the initial value of ${{\rm{\beta }}}_{\mathrm{GT}}={{\rm{\beta }}}_{{\rm{G}}}^{\mathrm{pre}}$), respectively. In other words, it is preferable to assume no prior information about ${{\rm{\beta }}}_{\mathrm{GT}}$, which aligns with the truth, than to assume incorrect prior information. Other initial values of ${{\rm{\beta }}}_{\mathrm{GT}}$ can be further explored to understand their impact on PRS prediction results in the future. Our real data analysis further shows that by focusing on M1, M3 and M5, PRS-PGx-TL substantially improves the predictive *p* value, and patient stratification performance. More specifically, PRS-PGx-TL improves the overall *R*^2^ by 3%–8%, increases the predictive −log10(*p* value) by 2–13 times, and enhances the differential treatment effect in patient stratification by 2.4–5.7 times. Top SNPs ranked by their absolute predictive effect sizes are generally consistent with the known knowledge, which makes biological sense.

Among M1, M3 and M5, M1 has the greatest improvement in overall *R*^2^ and *R*^2^ for prognostic (G) effect, while M3 and M5 have the smallest predictive *p* value and the largest conditional *R*^2^ for predictive (GTI) effect. The main difference between the M3 and M5 is that M3 updates both ${{\rm{\beta }}}_{{\rm{G}}}$ & ${{\rm{\beta }}}_{\mathrm{GT}}$ while M5 updates only ${{\rm{\beta }}}_{\mathrm{GT}}$ by fixing ${{\rm{\beta }}}_{{\rm{G}}}$. Compared with M3, M5 relies on a more stringent assumption by fixing ${{\rm{\beta }}}_{{\rm{G}}}$, which may not be necessarily the case in real data since the populations from disease cohort and the placebo arm of the PGx study may be different and their genetic architectures may be different (i.e., ${{\rm{\beta }}}_{{\rm{G}}}$ needs to be updated in such case). Therefore, when applying the proposed PRS-PGx-TL models in real-world clinical settings, the choice of model (M1 or M3) should align with the specific clinical objective: M1 is recommended for maximizing overall drug response prediction accuracy, while M3 is preferred for subgroup identification, patient stratification, and companion diagnostics (CDx) development. In addition, our simulations further show that as more information is shared between the base disease GWAS and target PGx data (i.e., as ${\rho }_{\mathrm{DT}}$ increases), the overall *R*^2^ for PRS-PGx-TL increases as well, which indicates that the information borrowed from the disease GWAS (base) does help the drug response prediction in PGx target data. The choice of baseline PRS-Dis models also affects the performance of the transfer learning algorithm. Compared with the Bayesian method (PRS-CS), sparser models (e.g., Lassosum) align with the TL framework better, especially when the proportion of causal SNPs is not that large. This is because sparser models filter a large number of SNPs with zero effects and thus significantly reduce the number of SNPs entering the gradient descent step. This indicates that the baseline PRS method may influence the transfer learning performance; thus, careful selection of the baseline PRS method may be required for good prediction results.

There are some limitations of the proposed PRS-PGx-TL methods and their applications to PGx data. First, our proposed PRS-PGx-TL method is primarily designed for pharmacogenomic (GWAS) data derived from randomized clinical trials (RCTs) featuring two arms (treatment and placebo). Considering the domain shift from disease space to PGx space, the disparity between disease GWAS and PGx GWAS can significantly impact transfer learning performance in real data analysis. Although our proposed TL-based framework and method hold great promise, the PRS prediction performance may not be good if the underlying genetic architectures are dramatically different (i.e., in terms of the prognostic effects, for example, if the control arm in PGx study is an active drug that is dramatically different from the disease population in the base cohort. In such cases, our method can still be applied, but its performance may vary). Second, an ideal PGx PRS combines genetic information with drug response data to predict an individuals’ response to a specific drug. The current framework and method do not consider some key drug-related information, such as drug targets, mechanism of action, pathways and gene-drug interaction networks in the feature (SNP) selection and parameter fine-tuning process. By leveraging the disease GWAS data with a related phenotype, incorporating such key drug information may help adapt the models to the specific drug response prediction task. Third, like most diseases and complex traits also have major environmental risk effects, drug response can be complex and usually influenced by multiple factors, including genetics, environment, and patient-specific characteristics, etc. Our current research work focuses only on genetic factors. One future direction may be to explore incorporating environmental factors (i.e., genotype by environment interaction) into PRS modeling. Fourth, accurate estimation of the genotype by treatment interaction effects usually requires sufficient sample size and power in the target PGx data. As PGx data needs to be split into training, validation, and testing in our PRS-PGx-TL PRS construction framework, the proposed framework and methods may not work well if the sample size of a target PGx study is too small. Fifth, the proposed methods mainly focus on continuous drug response clinical outcomes and the evaluation metric of mean square error. In the future, we may extend them to handling binary and survival drug response clinical outcomes, using appropriate metrics such as AUC-ROC, precision-recall curve, Harrell’s C-index, etc., to evaluate the transfer learning models. In Algorithm 1, we use the residuals of the original drug response, adjusted for covariates and treatment (**T**), when the drug response endpoint is a continuous variable. This saves computational cost in the iterative main algorithm because we don’t need to re-adjust for covariates and T in each iteration. If the drug response endpoint is a binary or time-to-event variable, our framework (Algorithm 1) still applies. In these cases, we’ll adjust for covariates and T within each iteration, rather than pre-calculating residuals as we do with continuous endpoints. Naturally, the loss function and the effect size iteration formula will change to accommodate the binary or time-to-event nature of the drug response (Y). Sixth, we choose not to compare PRS-PGx-TL with the direct (summary-statistics-level-based) PRS-PGx approach in our data analysis. To our knowledge, no publicly available PGx GWAS summary statistics include both genetic (G) and gene-treatment interaction (GTI) effect sizes. This limitation makes it impossible to apply this direct PRS-PGx approach in our real data analysis, even though some well-studied drug responses, like LDL-C’s response to statins, have publicly available PGx GWAS summary statistics from the treatment arm only in resources like the GWAS Catalog or PRS Catalog. A key area for future research is determining how to best construct PRS when the base cohort consists of PGx GWAS summary statistics (derived solely from a treatment arm) (along or not along with an independent disease GWAS summary statistics data) and the target/testing cohort is an independent, individual-level PGx GWAS dataset from two arms. Seventh, our PRS-PGx-TL method currently employs L2 regularization. It would be worthwhile to investigate extending it to L1 regularization, which would involve changing the current gradient descent algorithm to a coordinate descent algorithm. This algorithm utilizes closed-form soft-thresholding to update one parameter at a time^20^. In the future, we will evaluate this extension and its performance, particularly in scenarios with numerous irrelevant predictors. Eighth, we currently use a single parameter (*λ*) for both prognostic and predictive effects to simplify parameter tuning and reduce computational burden. In future studies, we can further investigate the effects of using separate hyperparameters for these two types of effects. Lastly, beyond the two-dimensional penalized gradient descent algorithm proposed in this research work, other transfer learning methods may also show promise for the PRS modeling with GxT interactions. For example, Bayesian methods^21^, neural networks^22^, and other optimization algorithms^16,23^, can effectively handle high-dimensional data and iteratively estimate both main effects and interaction effects. It is worthwhile to further compare PRS-PGx-TL with these methods in the future.

Transfer learning holds significant promise for advancing PRS research and improving its clinical utility. Unlike traditional PRS focusing on disease risk, a well-constructed/calibrated PGx PRS can be used to personalize drug therapy based on genetic makeup. The applications of our proposed methods may include not only drug response prediction and patient stratification (i.e., identifying patients who are likely to respond to a specific treatment or those at increased risk of adverse drug reactions), but also drug selection (i.e., identifying the most effective medications) and dose optimization (determining the optimal drug dose to minimize side effects and maximize efficacy). In summary, our proposed TL-based PRS method shows great value in improving drug response prediction and patient stratification and can help enhance precision medicine by using an individual’s genotype information to guide treatment.

## Methods

### Existing disease PRS methods

Many statistical methods have been proposed to construct PRS and predict complex traits^24–26^. These methods have been widely used for identifying individuals with high disease risk, and roughly fall into three categories: simple methods that ignore modeling linkage disequilibrium (LD) among SNPs such as Clumping and Thresholding (C + T) or Pruning + Thresholding (P + T); (2) machine learning methods such as Lassosum; and (3) Bayesian methods such as PRS-CS and PRS-CSx, etc. We refer to them as “disease PRS methods” in this paper. In our proposed transfer learning based PRS method PRS-PGx-TL, most of these disease PRS methods can be used as baseline methods to obtain the initial SNP weights from the large-scale disease GWAS base cohort. In this paper, three representative disease PRS methods were used as the baseline methods in our PGx studies: C + T (a simple method), Lassosum (a machine learning method), and PRS-CS (a Bayesian method). We give a brief introduction of each below.

1. The C + T method^27^ is a traditional method that uses two steps to select SNPs: (1) clumping, which removes SNPs in high linkage disequilibrium (LD) and keeps relatively independent SNPs, (2) *p* value thresholding, which retains significant SNPs while filtering SNPs using a series of GWAS *p* value cutoffs. C + T is an attractive PRS method since it is computationally efficient and straightforward to be implemented and interpreted. However, C + T only utilizes a portion of independent SNPs in constructing the PRS model and other SNPs and their LD information are not considered. Additionally, to choose the best *p* value cutoff for maximizing the prediction accuracy, C + T requires an external individual-level genotype dataset to evaluate different parameter values and choose the optimal one^25,27^. In other words, it does not work when there is only GWAS summary statistics available.
2. Lassosum is a frequentist PRS method utilizing penalized regression to shrink SNP effects^28^. It computes LASSO/Elastic Net estimates given summary statistics from GWAS, utilizing a reference panel to account for LD structure. As it takes the LD into consideration, it usually achieves improved predictive accuracy compared to C + T. Another important feature of Lassosum is that it provides a pseudo-validation version, which does not rely on independent validation data to do parameter tuning. The authors of Lassosum have also demonstrated that pseudo-validation can have prediction accuracy that is comparable to using an external validation dataset^28^.
3. PRS-CS uses a Bayesian framework with continuous shrinkage priors on SNP effect sizes^29^. In its Bayesian framework, it models effect sizes as random variables with prior distributions that adaptively shrink the effects towards zero, where the amount of shrinkage depends on the strength of the associations from GWAS. PRS-CS also utilizes both GWAS summary statistics and an external LD reference panel. These features make it robust to varying genetic architectures. Of note, PRS-CS also provides auto-version, which does not rely on independent validation data to do parameter tuning, so that it can be applied to large-scale studies where individual-level validation data is not available. The authors observed an average improvement of 48.16% and 38.62% in prediction accuracy across complex traits when comparing PRS-CS and PRS-CS auto-version with C + T^29^.

Together, these three methods represent a spectrum of disease PRS models, from traditionally simple models to more sophisticated models built on frequentist (machine learning) and Bayesian frameworks.

### PRS-PGx-TL

We propose the following model to jointly leverage the large-scale disease GWAS base data and PGx GWAS target data:

1 $$\begin{array}{rcl}{\bf{Y}}&=&{\bf{C}}{\boldsymbol{\delta }}+\mathop{\sum }\limits_{{\rm{j}}=1}^{{\rm{M}}}{{{\rm{\beta }}}_{\mathrm{jG}}{\bf{G}}}_{{\bf{j}}}+\mathop{\sum }\limits_{{\rm{j}}=1}^{{\rm{M}}}{{{\rm{\beta }}}_{\mathrm{jGT}}{\bf{G}}}_{{\bf{j}}}* {\bf{T}}{\boldsymbol{+}}{\boldsymbol{\epsilon }}\\&=& {\bf{C}}{\boldsymbol{\delta }}+{\bf{G}}{{\boldsymbol{\beta }}}_{{\rm{G}}}+\left({\bf{G}}\times {\bf{T}}\right){{\boldsymbol{\beta }}}_{\mathrm{GT}}+{\boldsymbol{\epsilon }}\end{array},$$

where ${\bf{Y}}$ denotes the drug response, and ${\bf{C}}$ is a matrix of non-genetic covariate information. ${\bf{G}}$ denotes the genotype matrix, and ${\bf{T}}$ denotes the treatment group. ${{\boldsymbol{\beta }}}_{{\rm{G}}},{{\boldsymbol{\beta }}}_{{\rm{GT}}}$ denote the true prognostic and predictive effect sizes in the target group, assumed to be unknown. Combining the effect sizes ${{\boldsymbol{\beta }}}_{{\rm{G}}},{{\boldsymbol{\beta }}}_{{\rm{GT}}}$, we have

2 $${\bf{Y}}={\bf{C}}{\boldsymbol{\delta }}+{\bf{Xb}}+{\boldsymbol{\epsilon }},$$

where ${\bf{X}}={\boldsymbol{[}}{\bf{G}}\,\left({\bf{G}}\times {\bf{T}}\right)]$, ${\bf{b}}=\left[{{\boldsymbol{\beta }}}_{{\rm{G}}}\,{{\boldsymbol{\beta }}}_{{\rm{GT}}}\right].$ The overall goal is to minimize the loss function:

3 $${\rm{loss}}={\left({\bf{Y}}-{\bf{C}}{\boldsymbol{\delta }}-{\bf{Xb}}\right)}^{{\rm{T}}}\left({\bf{Y}}-{\bf{C}}{\boldsymbol{\delta }}-{\bf{Xb}}\right)+{\rm{\lambda }}{{\bf{b}}}^{{\rm{T}}}{\bf{b}},$$

where ${\rm{\lambda }}$ is the penalization or regularization parameter, which controls the model complexity and prevents overfitting.

To incorporate the information from the disease GWAS (base) data or jointly analyze the disease GWAS (base) data and the PGx GWAS (target) data, a two-dimensional penalized gradient descent algorithm is further proposed to update ${\bf{b}}$, with initial values ${{\bf{b}}}^{0}=[{{\boldsymbol{\beta }}}_{{\rm{G}}}^{{\rm{pre}}}\,{{\boldsymbol{\beta }}}_{{\rm{GT}}}^{{\rm{pre}}}]$ calculated by the model that was pre-trained using disease GWAS:

4 $$\frac{\partial {\rm{loss}}}{\partial {\bf{b}}}=-{2{\bf{X}}}^{{\rm{T}}}\left({\bf{Y}}-{\bf{C}}{\boldsymbol{\delta }}-{\bf{Xb}}\right)+2{\rm{\lambda }}{\bf{b}},$$

5 $${{\bf{b}}}^{\left({\rm{r}}+1\right)}={{\bf{b}}}^{\left({\rm{r}}\right)}-{\rm{\eta }}\frac{\partial {\rm{loss}}}{\partial {\bf{b}}},$$

6 $${{\bf{b}}}^{\left({\rm{r}}+1\right)}=\left(1-2{\rm{\eta }}{\rm{\lambda }}\right){{\bf{b}}}^{\left({\rm{r}}\right)}+2{\rm{\eta }}{{\bf{X}}}^{{\rm{T}}}\left({\bf{Y}}-{\bf{C}}{\boldsymbol{\delta }}-{\bf{Xb}}\right),$$

where ${\rm{\eta }}$ is the learning rate. For simplicity, we define a new parameter ${{\rm{\lambda }}}^{{\rm{new}}}\,:=2{\rm{\eta }}{\rm{\lambda }}$ and choose it from {0,0.5,0.99} to prevent the term $\left(1-{{\rm{\lambda }}}^{{\rm{new}}}\right)$ from being negative, which would flip the sign of ${{\bf{b}}}^{\left({\rm{r}}\right)}$ at each iteration and cause instability in the gradient descent updates. We also define ${{\rm{\eta }}}^{{\rm{new}}}\,:=2{\rm{\eta }}$ to absorb “2”. The new update rule then becomes:

7 $${{\bf{b}}}^{\left({\rm{r}}+1\right)}=\left(1-{{\rm{\lambda }}}^{{\rm{new}}}\right){{\bf{b}}}^{\left({\rm{r}}\right)}+{{\rm{\eta }}}^{{\rm{new}}}{{\bf{X}}}^{{\rm{T}}}\left({\bf{Y}}-{\bf{C}}{\boldsymbol{\delta }}-{\bf{Xb}}\right).$$

The learning rate ${{\rm{\eta }}}^{{\rm{new}}}$ (denote as ${\rm{\eta }}$ below), regularization parameter ${{\rm{\lambda }}}^{{\rm{new}}}\,$(denote as ${\rm{\lambda }}$ below), and number of best iteration ${\rm{r}}$ can be tuned and optimized based on the validation dataset (“inner layer”). The learning rate ${\rm{\eta }}$ controls the step size taken towards minimizing the loss function during optimization. To balance computational cost with effective optimization, we followed Zhao et al.’s suggestion^30^, selecting ${\rm{\eta }}$ from a small grid of values: $\{\frac{\mathrm{1,10,50,100}}{m}\}$, where m is the number of variants with non-zero effects in ${{\boldsymbol{\beta }}}_{{\rm{G}}}^{{\rm{pre}}}$. The regularization parameter ${\rm{\lambda }}\in [0,\,1)$ controls the strength of shrinkage applied to the effect sizes. Our chosen values for *λ* $\{\mathrm{0,0.5,0.99}\}$, correspond to no shrinkage, a medium degree of shrinkage, and a large degree of shrinkage, respectively. In addition, the parameter $n.{iter}$, the total number of iterations, is set to 30. Within these 30 iterations, we select the iteration that yields the largest ${R}^{2}$ as the best. We cap iterations at 30 to prevent overfitting since too many iteration steps would result in overfitting^30^ and the optimal iteration number (with the largest overall *R*^2^ or conditional *R*^2^) typically occurs early in the iterative process (Fig. S10).

The PRS-PGx-TL is fit using the two-dimensional penalized gradient descent algorithm. With initial values ${{\boldsymbol{\beta }}}_{{\rm{G}}}^{{\rm{pre}}}$ from the baseline disease PRS methods, we use a gradient descent algorithm to update the prognostic and predictive effects ${{\boldsymbol{\beta }}}_{{\rm{G}}},{{\boldsymbol{\beta }}}_{{\rm{GT}}}$ simultaneously with the L2 penalty loss function. Of note, L2 regularization was used due to (1) the simplicity to get a closed-form update in the gradient descent algorithm to update all parameters simultaneously, (2) our method is built upon certain baseline disease PRS methods, which allows us to flexibly choose L1-based PRS models as baseline methods. In this case, our method can start from the selected features from baseline models even though itself is L2-based. After applying the four-fold cross-validation to select the optimal parameters, the final weights of PRS-PGx-TL can be outputted. The algorithm was summarized in the following Algorithm 1.

#### Algorithm 1

Two-dimensional penalized gradient descent algorithm

**Preparing pre-trained model:** apply baseline disease PRS methods to the disease GWAS summary statistics (base cohort) to get ${{\boldsymbol{\beta }}}_{{\rm{G}}}^{{\rm{pre}}}$.

**Input:** PRS weights ${{\boldsymbol{\beta }}}_{{\rm{G}}}^{{\rm{pre}}}$, individual-level PGx data ${\bf{X}}=\left[{\bf{G}}\,\left({\bf{G}}\times {\bf{T}}\right)\right]$ and drug response ${\bf{Y}}$. In the presence of non-genetic covariates such as sex, age, and top principal components etc., ${\bf{Y}}$ is the residuals of the original drug response by first adjusting covariates and ${\bf{T}}$. Otherwise, ${\bf{Y}}$ is the residuals of the original drug response after adjusting ${\bf{T}}$.

Hyperparameters: learning rate ${\rm{\eta }}$, regularization parameter ${\rm{\lambda }}$, number of iterations parameter ${\rm{r}}$.

Initialization: ${\rm{\eta }}$ from $\{\frac{1,10,50,100}{{\rm{m}}}\}$ where m is the number of variants with non-zero effects in ${{\boldsymbol{\beta }}}_{{\rm{G}}}^{{\rm{pre}}}$, ${\rm{\lambda }}$ from $\{0,0.5,0.99\}$, ${\rm{n}}.{\rm{iter}}=30$, ${{\bf{b}}}^{0}=\left[{{\boldsymbol{\beta }}}_{{\rm{G}}}^{0}{{\boldsymbol{\beta }}}_{{\rm{GT}}}^{0}\right]$ where ${{\boldsymbol{\beta }}}_{{\rm{G}}}^{0}={{\boldsymbol{\beta }}}_{{\rm{G}}}^{{\rm{pre}}}$, ${{\boldsymbol{\beta }}}_{{\rm{GT}}}^{0}={\bf{0}}$ or ${{\boldsymbol{\beta }}}_{{\rm{G}}}^{{\rm{pre}}}$.

**Main algorithm:**

**while** r < n.iter **do**

  Update $${{\bf{b}}}^{({\rm{r}}+1)}=(1-{\rm{\lambda }}){{\bf{b}}}^{({\rm{r}})}+{\rm{\eta }}{{\bf{X}}}^{{\rm{T}}}\left({\bf{Y}}-{\bf{X}}{{\bf{b}}}^{\left({\rm{r}}\right)}\right)$$,

    ${{\boldsymbol{\beta }}}_{{\rm{G}}}^{({\rm{r}})}$ could be fixed at ${{\boldsymbol{\beta }}}_{{\rm{G}}}^{0}$ or updated in each iteration here.

**end**

**Parameter tuning:**

Divide the training dataset into 4 folds for cross-validation

  In each fold, train the model using the main algorithm on 3 folds, keep ${{\bf{b}}}^{(1)},\ldots ,{{\bf{b}}}^{({\rm{n}}.{\rm{iter}})}$ for each ${\rm{\eta }}$ and ${\rm{\lambda }}$ combination, and validate on the remaining fold:

    Calculate $${\bf{PR}}{{\bf{S}}}_{{\bf{G}}}=$$ $${\bf{G}}{{\boldsymbol{\beta }}}_{{\rm{G}}}$$, $${\bf{PR}}{{\bf{S}}}_{{\bf{GT}}}=$$ $${\bf{G}}{{\boldsymbol{\beta }}}_{{\rm{GT}}}$$,

    Calculate the ${{\bf{R}}}^{{\bf{2}}}$ for the model ${\bf{Y}}{\boldsymbol{ \sim }}{{\bf{PRS}}}_{{\bf{G}}}{\boldsymbol{+}}{{\bf{PRS}}}_{{\bf{GT}}}{\boldsymbol{\times }}{\bf{T}}$ or

      the conditional ${{\bf{R}}}^{{\bf{2}}}$ for the model ${\bf{Y}}{\boldsymbol{ \sim }}{\bf{PR}}{{\bf{S}}}_{{\bf{GT}}}{\boldsymbol{\times }}{\bf{T}}|{\bf{PR}}{{\bf{S}}}_{{\bf{G}}}$,

  Calculate the average performance (R^2^ or conditional R^2^) across all four folds to obtain the cross-validation score for each combination of hyperparameters.

Select the best combination of ${\rm{\eta }},{\rm{\lambda }},{\rm{r}}$ based on the best cross-validation score.

**Final Training:**

Combine all data (training and validation sets), re-apply the main algorithm with the best parameters ${\rm{\eta }},{\rm{\lambda }},{\rm{r}}$.

**Output:** $$\hat{{\bf{b}}}=[{\hat{{\boldsymbol{\beta }}}}_{{\rm{G}}}\,{\hat{{\boldsymbol{\beta }}}}_{{\rm{GT}}}]$$.

### Simulation studies

We conducted extensive simulation studies to benchmark the performance of our proposed transfer-learning-based PRS-PGx-TL method. PRS-PGx-TL with six different implementation strategies were compared with disease PRS approaches (i.e., C + T, Lassosum, and PRS-CS).

We first simulated the true effect sizes in the base and target cohorts simultaneously, following the spike-and-slab distribution:

8 $$\left(\begin{array}{c}{{\rm{\mu }}}_{{\rm{j}}}\\ {{\rm{\beta }}}_{{\rm{j}}}\\ {{\rm{\alpha }}}_{{\rm{j}}}\end{array}\right)|{{\rm{R}}}_{{\rm{j}}}=\left\{\begin{array}{cc}{\rm{MVN}}\left(0,\Sigma \right) & ,\,{{\rm{R}}}_{{\rm{j}}}=1\\ 0 & ,\,{{\rm{R}}}_{{\rm{j}}}=0\end{array}\right.,{\rm{where\; }}{{\rm{R}}}_{{\rm{j}}}\sim {\rm{Bernoulli}}\left({\rm{p}}\right),{\rm{and\; \Sigma }}=\frac{{{\rm{h}}}_{{\rm{T}}}^{2}}{{\rm{mp}}}\Omega ,$$

where ${{\rm{\mu }}}_{{\rm{j}}}$ denotes the effect size of SNP ${\rm{j}}$ in the base cohort of a disease GWAS and ${{\rm{\beta }}}_{{\rm{j}}}$ and ${{\rm{\alpha }}}_{{\rm{j}}}$ denote the prognostic and predictive effect sizes of SNP ${\rm{j}}$ (i.e., ${{\rm{\beta }}}_{{\rm{jG}}}$ and ${{\rm{\beta }}}_{{\rm{jGT}}}$) in the target cohort of a PGx GWAS, respectively. ${\rm{p}}$ is the proportion of causal variants, ${{\rm{h}}}_{{\rm{T}}}^{2}$ is the heritability of the trait in the base cohort, and ${\rm{m}}$ is the number of causal SNPs. $\Omega$ is the correlation matrix, which was set as

9 $$\Omega |{{\rm{\rho }}}_{{\rm{E}}},{{\rm{\rho }}}_{{\rm{DT}}}=\left[\begin{array}{ccc}1 & {{\rm{\rho }}}_{{\rm{DT}}} & {{\rm{\rho }}}_{{\rm{DT}}}\times {{\rm{\rho }}}_{{\rm{E}}}\\ {{\rm{\rho }}}_{{\rm{DT}}} & 1 & {{\rm{\rho }}}_{{\rm{E}}}\\ {{\rm{\rho }}}_{{\rm{DT}}}\times {{\rm{\rho }}}_{{\rm{E}}} & {{\rm{\rho }}}_{{\rm{E}}} & 1\end{array}\right],$$

where ${{\rm{\rho }}}_{{\rm{DT}}}$ is the prognostic effect correlation between disease GWAS in the base cohort and PGx GWAS in the target cohort. ${{\rm{\rho }}}_{{\rm{E}}}$ is the correlation between prognostic and predictive effects in the target cohort.

After the true effect sizes in both base (${{\rm{\mu }}}_{{\rm{j}}}$) and target (${{\rm{\beta }}}_{{\rm{j}}},\,{{\rm{\alpha }}}_{{\rm{j}}}$) cohorts were simulated, we further simulated disease GWAS summary statistics in the base cohort. Standard deviations (${{\rm{s}}}_{{\rm{j}}}$) were extracted from the Global Lipids Genetics Consortium (GLGC) (https://csg.sph.umich.edu/willer/public/glgc-lipids2021/) GWAS summary statistics for the LDL-C trait. We assumed effect sizes from the summary statistics followed a normal distribution ${\hat{{\rm{\mu }}}}_{{\rm{j}}}|{{\rm{\mu }}}_{{\rm{j}}}\sim {\rm{N}}({{\rm{\mu }}}_{{\rm{j}}},{{\rm{s}}}_{{\rm{j}}}^{2})$. *p* values were calculated as ${{\rm{p}}}_{{\rm{j}}}=2\times (1-{\rm{\psi }}(|{\hat{{\rm{\mu }}}}_{{\rm{j}}}/{{\rm{s}}}_{{\rm{j}}}|))$, where ${\rm{\psi }}$ denotes the cumulative density function of a standard normal distribution. We then simulated individual-level PGx data in the target cohort. We set ${{\rm{\alpha }}}^{* }={\rm{\gamma }}* {\rm{\alpha }}$, where ${\rm{\gamma }}$ is a scale factor that quantifies different magnitudes for the prognostic (${\rm{\beta }}$) and predictive (${\rm{\alpha }}$) effects. We used the real genotype matrix (${\rm{G}}$) from chromosome 19 of the IMPROVE-IT PGx GWAS data, which includes 20,854 SNPs in total. The drug response was generated following the model:

10 $${\rm{Y}}={{\rm{\beta }}}_{{\rm{T}}}{\rm{T}}+{\rm{G}}{\rm{\beta }}+\left({\rm{G}}\times {\rm{T}}\right){{\rm{\alpha }}}^{* }+{\rm{\epsilon }},{\rm{\epsilon }}\sim {\rm{N}}\left(0,{{\rm{\sigma }}}^{2}\right),$$

where the treatment variable was simulated as T ∼ Bernoulli(0.5) and ${{\rm{\sigma }}}^{2}$ was determined by the heritability ${{\rm{h}}}_{{\rm{D}}}^{2}$. In Table S1, we provide details of the parameter setup for $({\rm{p}},\,{{\rm{h}}}_{{\rm{T}}}^{2},\,{{\rm{\rho }}}_{{\rm{DT}}},\,{{\rm{\rho }}}_{{\rm{E}}},\,{{\rm{\beta }}}_{{\rm{T}}},\,{{\rm{h}}}_{{\rm{D}}}^{2},{\rm{\gamma }})$. After ${\rm{PR}}{{\rm{S}}}_{{\rm{G}}}$ and ${\rm{PR}}{{\rm{S}}}_{{\rm{GT}}}$ were calculated, we further evaluated the performance of different PRS methods following the model:

11 $${{\rm{Y}}}^{{\prime} }\sim {\rm{PR}}{{\rm{S}}}_{{\rm{G}}}+{\rm{PR}}{{\rm{S}}}_{{\rm{GT}}}\times {\rm{T}},$$

where ${{\rm{Y}}}^{{\prime} }$ is the residual after adjusting T term. We introduced multiple evaluation criteria, including overall prediction accuracy ${R}^{2}$, partial ${R}^{2}$ explained by the ${\rm{PR}}{{\rm{S}}}_{{\rm{G}}}$ term, partial ${R}^{2}$ explained by the $\mathrm{PR}{{\rm{S}}}_{\mathrm{GT}}\times T$ term conditional on ${\rm{PR}}{{\rm{S}}}_{{\rm{G}}}$, ${\rm{PR}}{{\rm{S}}}_{{\rm{GT}}}$-by-treatment interaction *p* value, and average treatment effects in different PRS quantiles.

### Application to IMPROVE-IT PGx GWAS data

We applied disease PRS methods (C + T, Lassosum, PRS-CS) and the PRS-PGx-TL method with different implementation strategies to predict the drug response (LDL-C log-fold change at 1-month) from the IMPROVE-IT PGx GWAS. IMPROVE-IT is a phase 3b, multicenter, double-blind, randomized study to establish the clinical benefit and safety of Vytorin (Ezetimibe/Simvastatin combined therapy: 10 mg + 40 mg) versus Simvastatin monotherapy (40 mg) in high-risk subjects (registry name: ClinicalTrials.gov, registration number: NCT00202878, and the date of registration: September 13, 2005)^31^. The ethics committee at each participating center approved the protocol and amendments^31^. The IMPROVE-IT trial was carried out in accordance with the Declaration of Helsinki, current guidelines on Good Clinical Practices and local ethical and legal requirements^31^. All participants provided voluntary written informed consent before trial entry^31^. A completed CONSORT checklist of IMPROVE-IT trial is provided in Table S5. The details of the endpoint, genotyping, genotype QC and imputation for this GWAS analyses are introduced elsewhere^19^. After GWAS QC and SNP imputation, there were 9,407,967 variants and 6502 subjects remaining. The subjects were further filtered down to 5661 subjects for the GWAS analyses by excluding subjects who had a cardiovascular event prior to month 1, since cardiovascular events prior to this time point may affect LDL-C in a manner unrelated to treatment. After matching SNPs with GLGC data (used as disease GWAS summary statistics) and 1000 Genomes (1000 G) data (used as the reference panel), a total of 1,109,919 SNPs were included for analysis of the LDL-C drug response. Several variables were included as covariates in the analysis model, which included age, gender, prior lipid-lowering (PLL) therapy, early glycoprotein IIb/IIIa inhibition in non-ST-segment elevation acute coronary syndrome (EARLY ACS) trial, high-risk ACS diagnosis, baseline LDL-C level, and five top principal components^4,19^.

For disease PRS methods, we used the LDL-C disease GWAS summary statistics from GLGC to construct the PRS. For PRS-PGx-TL, we leveraged both the GLGC summary statistics and the IMPROVE-IT PGx data in the PRS construction. Specifically, we first applied the disease PRS methods to the GLGC summary statistics and obtained the SNP weights ${{\boldsymbol{\beta }}}_{{\rm{G}}}^{{\rm{pre}}}$. We then utilized ${{\boldsymbol{\beta }}}_{{\rm{G}}}^{{\rm{pre}}}$ as initial values for the two-dimensional penalized gradient descent algorithm. When applying PRS-PGx-TL methods, we used nested cross-validation procedure as described in section “Results”, and the final PRS for all the patients in the IMPROVE-IT PGx data would be obtained. Finally, we assessed the prediction performance using the same criteria as those in the simulations (overall prediction accuracy ${R}^{2}$, conditional ${R}^{2}$, ${\rm{PR}}{{\rm{S}}}_{{\rm{GT}}}$-by-treatment interaction *p* value, and average treatment effects in different PRS quantiles for patient stratification evaluation).

## Supplementary information

Supplementary Information

## Data Availability

GWAS summary statistics from the Global Lipids Genetics Consortium (GLGC): https://csg.sph.umich.edu/willer/public/glgc-lipids2021/. MSD’s data sharing policy, including restrictions, is available at http://engagezone.msd.com/ds_documentation.php. Requests for access to the GWAS summary statistics results from this IMPROVE_IT clinical study data can be submitted through the EngageZone site or via email to dataaccess@merck.com. 1000 Genomes (1KG) Phase 3 data: http://csg.sph.umich.edu/abecasis/mach/download/1000G.Phase3.v5.html. Lassosum: https://github.com/tshmak/lassosum. PRS-CS: https://github.com/getian107/PRScs.

## Code availability

Our PRS-PGx-TL data analysis pipeline and PRSPGxTL R package are implemented and freely available at https://github.com/YoushuCheng/PRS-PGx-TL and https://github.com/wjzhong/PRSPGxTL, respectively.
