# Supplementary material for: Improving polygenic risk score based drug response prediction using transfer learning
Source: NPJ Genom Med. 2025 Nov 21;10:74. doi: 10.1038/s41525-025-00528-x (PMC12638960; doi:10.1038/s41525-025-00528-x)
Supplement: Supplementary file 1 — Supplementary Information [file 41525_2025_528_MOESM1_ESM.pdf]

Supplementary Materials for

Improving Polygenic Risk Score Based Drug Response Prediction Using Transfer Learning

Youshu Cheng[1,2#], Song Zhai[2#], Wujuan Zhong[2], Rachel Marceau West[2], Judong Shen[2*]

[1]Department of Biostatistics, Yale University, New Haven, CT 06520, USA

[2]Biostatistics and Research Decision Sciences, Merck & Co., Inc., Rahway, NJ 07065, USA

# These authors contributed equally to this work

* To whom correspondence should be addressed

Correspondence: judong.shen@merck.com

This file includes:

Supplementary Figures S1-S10

Supplementary Tables S1-S5

Supplementary Figures

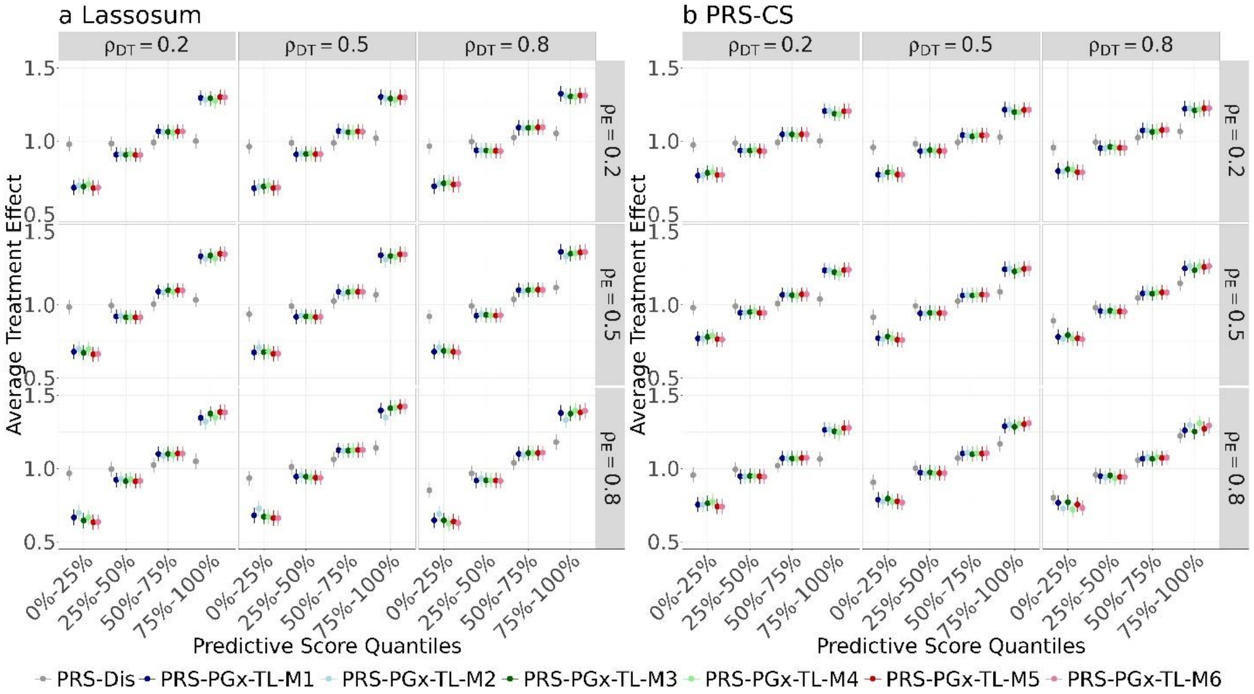

Figure S1 Simulation based patient stratification comparisons between disease PRS methods and PRS-PGx-TL method with different implementation strategies (M1 – M6) for (a) Lassosum, and (b) PRS-CS. $\mathbf{H_D^2 = 0.3}$, $\boldsymbol{\gamma = 1}$, **pcausal = 0.01**, $\boldsymbol{\rho_{DT}} \in \{\mathbf{0.2, 0.5, 0.8}\}$, $\boldsymbol{\rho_E} \in \{\mathbf{0.2, 0.5, 0.8}\}$. The graph was created with the open-source ggplot2 R package (https://ggplot2.tidyverse.org).

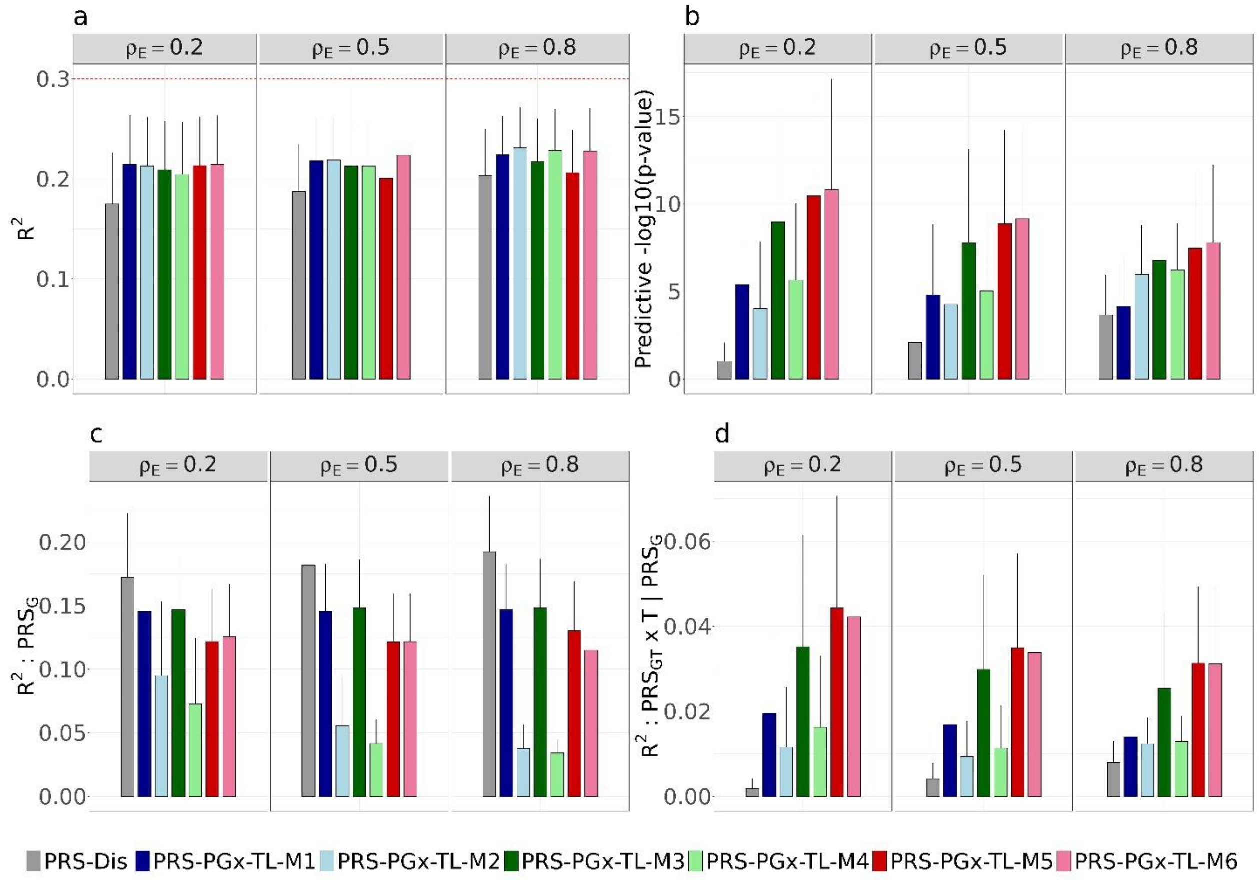

Figure S2 Simulation results based on C+T baseline disease PRS method in terms of (a) overall $\mathbf{R^2}$, (b) predictive p-value, (c) partial $\mathbf{R^2}$ explained by the $\mathbf{PRS_G}$ term, and (d) partial $\mathbf{R^2}$ explained by the $\mathbf{PRS_{GT}} \times \mathbf{T}$ term (conditional on $\mathbf{PRS_G}$). $\mathbf{H_D^2 = 0.3}$, $\boldsymbol{\gamma} = \mathbf{1}$, **pcausal = 0.01**, $\boldsymbol{\rho_{DT}} = \mathbf{1}$, $\boldsymbol{\rho_E} \in \{\mathbf{0.2, 0.5, 0.8}\}$ **.** The graph was created with the open-source ggplot2 R package (https://ggplot2.tidyverse.org).

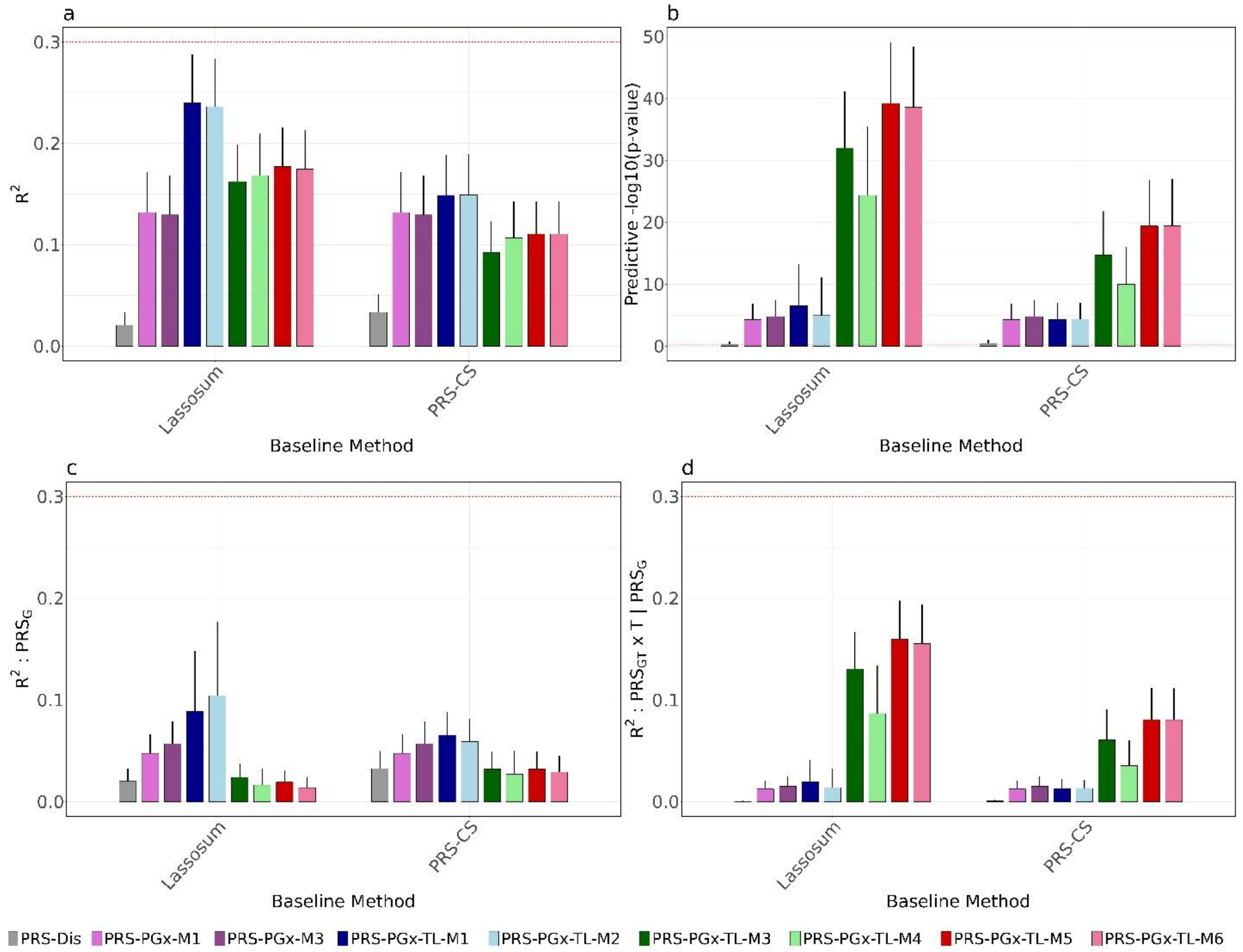

Figure S3 Simulation based performance comparisons between disease PRS methods (Lassosum, and PRS-CS), PRS-PGx method (the initial values $\boldsymbol{\beta_G}$ and $\boldsymbol{\beta_{GT}}$ start from 0), PRS-PGx-TL method with different implementation strategies (M1 – M6). Methods are compared in terms of (a) overall $\mathbf{R^2}$, (b) predictive p-value, (c) partial $\mathbf{R^2}$ explained by the $\mathbf{PRS_G}$ term, and (d) partial $\mathbf{R^2}$ explained by the $\mathbf{PRS_{GT}} \times \mathbf{T}$ term (conditional on $\mathbf{PRS_G}$). $\mathbf{H_D^2 = 0.3}$, $\boldsymbol{\gamma = 1}$, $\mathbf{pcausal = 0.01}$, $\boldsymbol{\rho_{DT} = 0.5}$, $\boldsymbol{\rho_E = 0.5}$. The graph was created with the open-source ggplot2 R package (https://ggplot2.tidyverse.org).

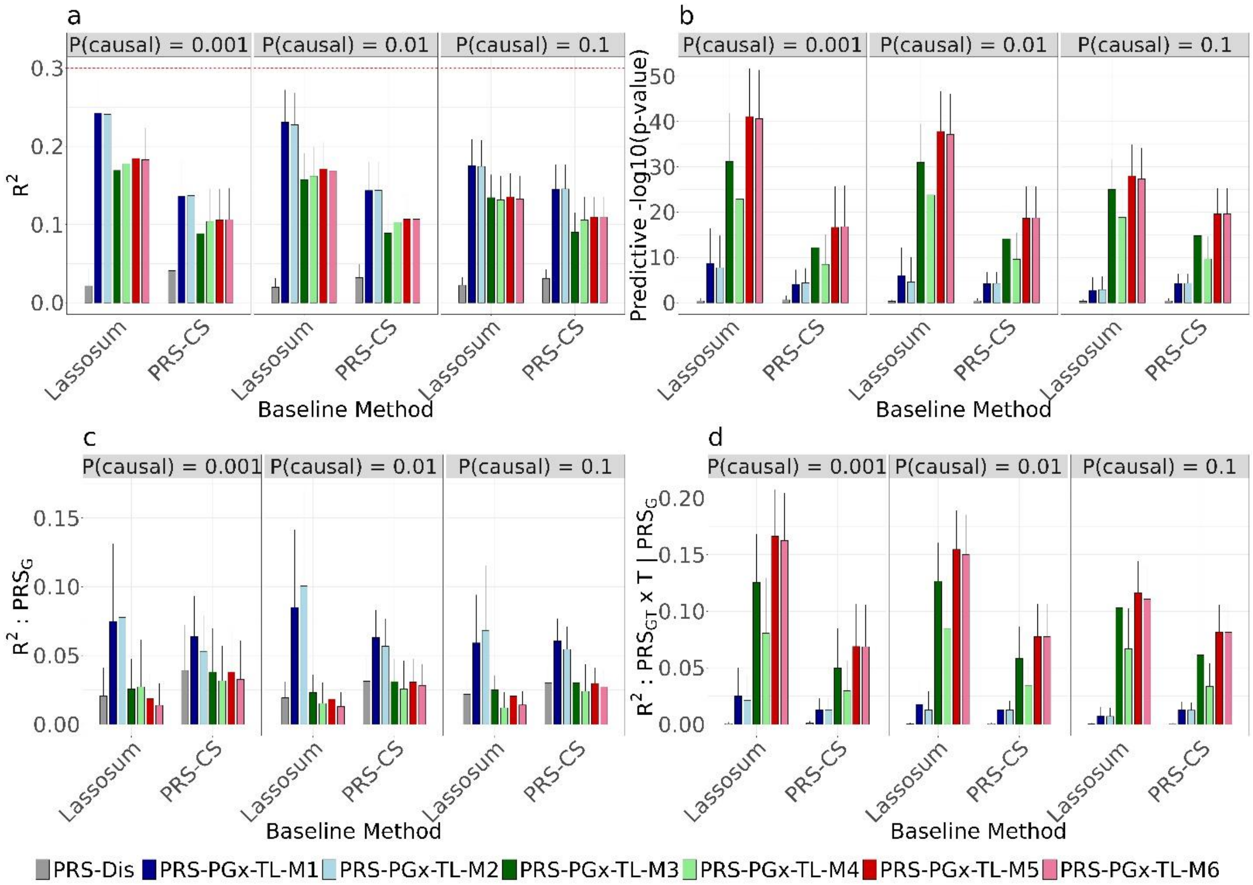

Figure S4 Simulation based performance comparisons between disease PRS methods (Lassosum, and PRS-CS) and PRS-PGx-TL method with different implementation strategies (M1 – M6). Methods are compared in terms of (a) overall $\mathbf{R^2}$, (b) predictive p-value, (c) partial $\mathbf{R^2}$ explained by the $\mathbf{PRS_G}$ term, and (d) partial $\mathbf{R^2}$ explained by the $\mathbf{PRS_{GT}} \times \mathbf{T}$ term (conditional on $\mathbf{PRS_G}$). $\mathbf{H_D^2 = 0.3}$, $\boldsymbol{\gamma} = \mathbf{1}$, $\mathbf{pcausal} \in \{\mathbf{0.001}, \mathbf{0.01}, \mathbf{0.1}\}$, $\boldsymbol{\rho}_{\mathbf{DT}} = \mathbf{0.5}$, $\boldsymbol{\rho}_{\mathbf{E}} = \mathbf{0.5}$. The graph was created with the open-source ggplot2 R package (https://ggplot2.tidyverse.org).

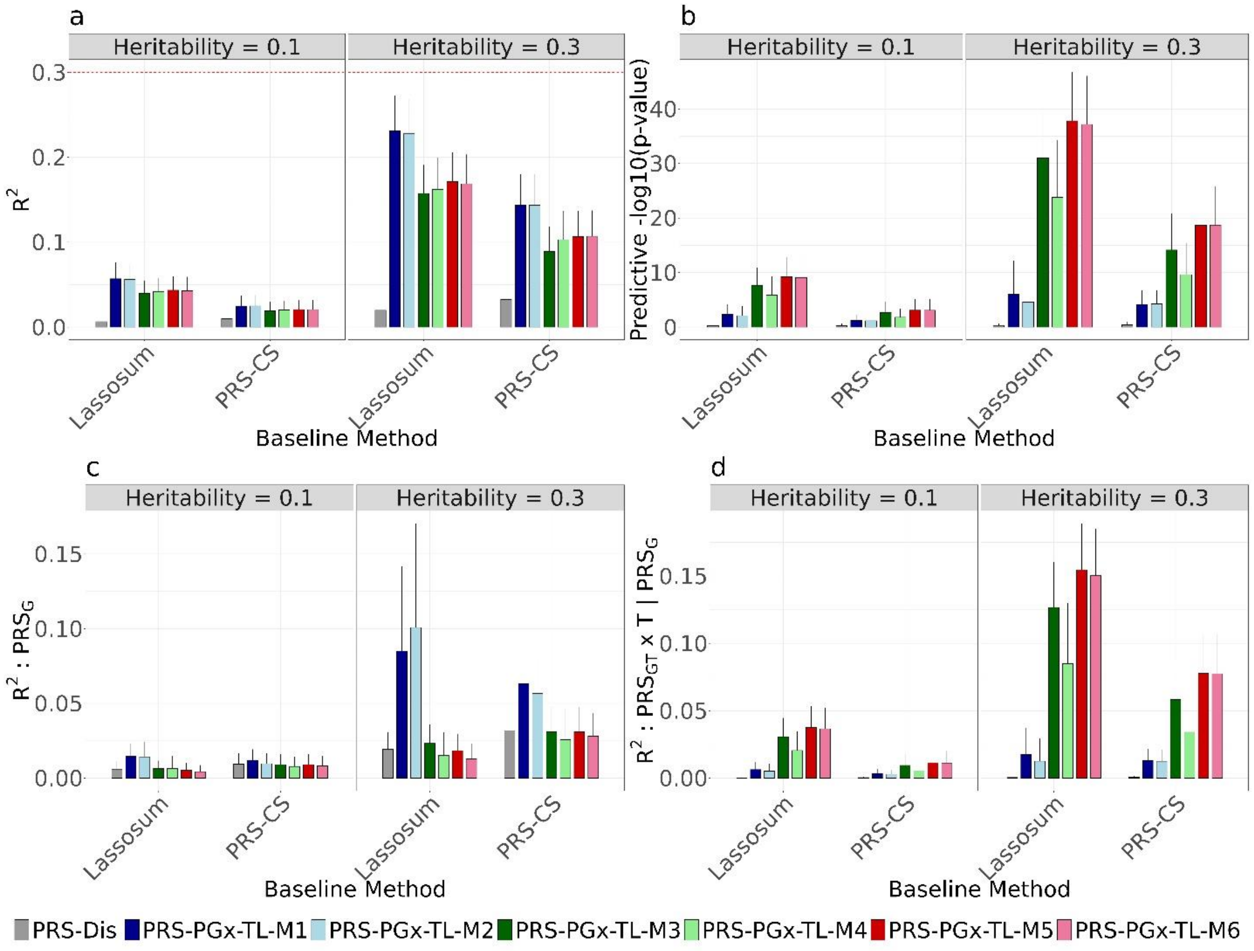

Figure S5 Simulation based performance comparisons between disease PRS methods (Lassosum, and PRS-CS) and PRS-PGx-TL method with different implementation strategies (M1 – M6). Methods are compared in terms of (a) overall $\mathbf{R^2}$, (b) predictive p-value, (c) partial $\mathbf{R^2}$ explained by the $\mathbf{PRS_G}$ term, and (d) partial $\mathbf{R^2}$ explained by the $\mathbf{PRS_{GT}} \times \mathbf{T}$ term (conditional on $\mathbf{PRS_G}$). $\mathbf{H_D^2} \in \{\mathbf{0.1}, \mathbf{0.3}\}$, $\boldsymbol{\gamma} = \mathbf{1}$, **pcausal = 0.01**, $\boldsymbol{\rho}_{\mathbf{DT}} = \mathbf{0.5}$, $\boldsymbol{\rho}_{\mathbf{E}} = \mathbf{0.5}$. The graph was created with the open-source ggplot2 R package (https://ggplot2.tidyverse.org).

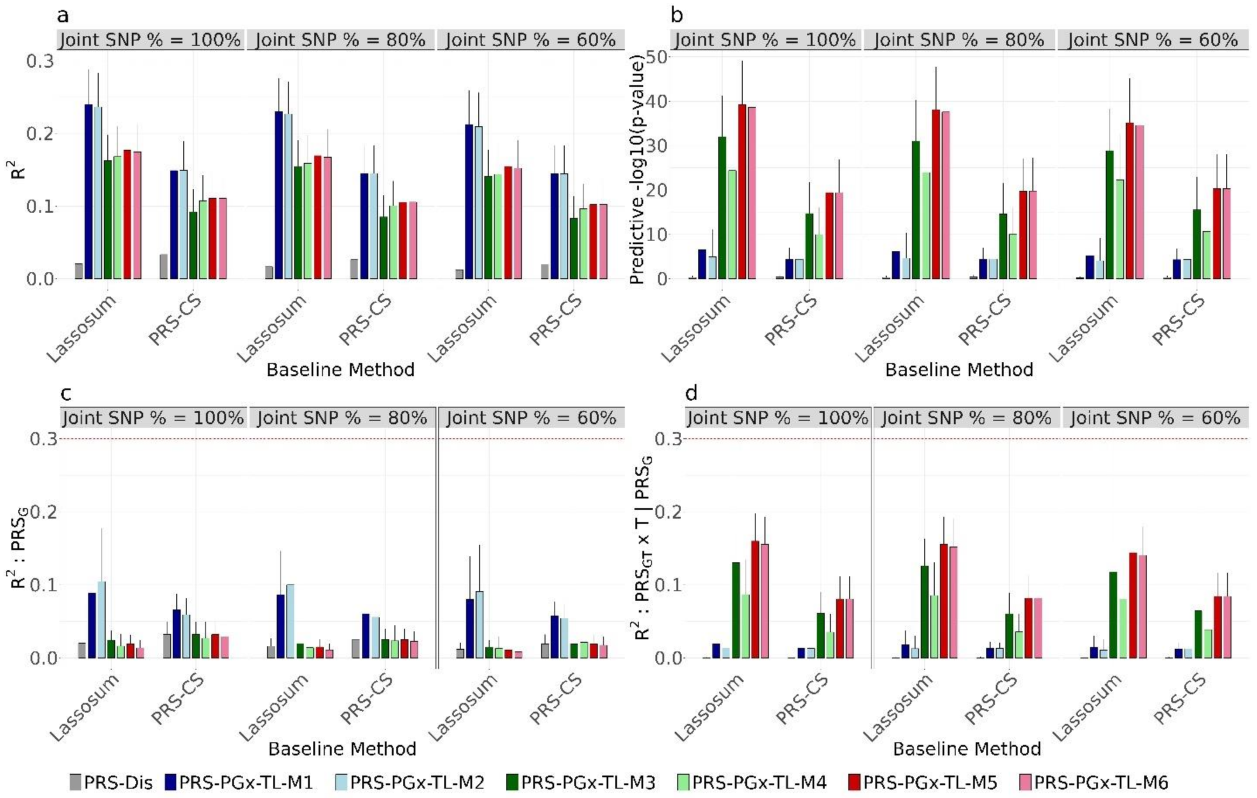

Figure S6 Simulation based performance comparisons between disease PRS methods (Lassosum, and PRS-CS) and PRS-PGx-TL method with different implementation strategies (M1 – M6). Methods are compared in terms of (a) overall $\mathbf{R^2}$, (b) predictive p-value, (c) partial $\mathbf{R^2}$ explained by the $\mathbf{PRS_G}$ term, and (d) partial $\mathbf{R^2}$ explained by the $\mathbf{PRS_{GT}} \times \mathbf{T}$ term (conditional on $\mathbf{PRS_G}$). $\mathbf{H_D^2 = 0.3}$, $\boldsymbol{\gamma} = \mathbf{1}$, **pcausal** $= \mathbf{0.01}$, $\boldsymbol{\rho}_{\mathbf{DT}} = \mathbf{0.5}$, $\boldsymbol{\rho}_{\mathbf{E}} = \mathbf{0.5}$, **percentage of joint causal variants on both disease and drug response** $\in \{\mathbf{100\%, 80\%, 60\%}\}$. The graph was created with the open-source ggplot2 R package (https://ggplot2.tidyverse.org).

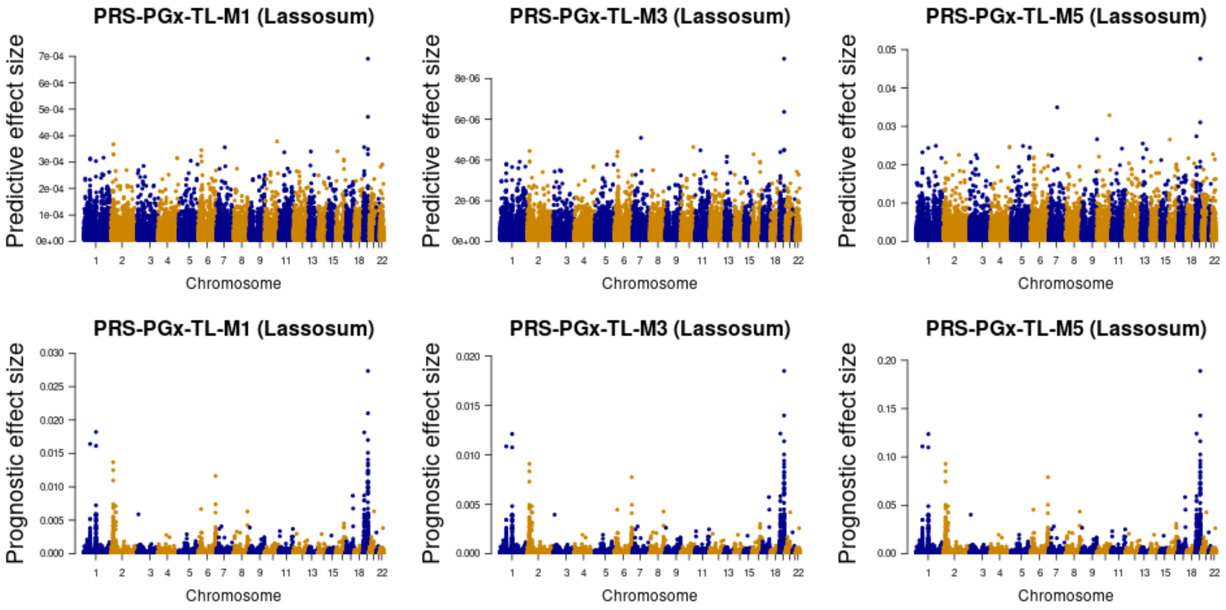

Figure S7 Manhattan plots of prognostic and predictive effect sizes for PRS-PGx-TL-M1/M3/M5 using Lassosum as the baseline method from the PRS analysis of the IMPROVE-IT PGx GWAS data. The graph was created with the open-source qqman R package (https://github.com/stephenturner/qqman).

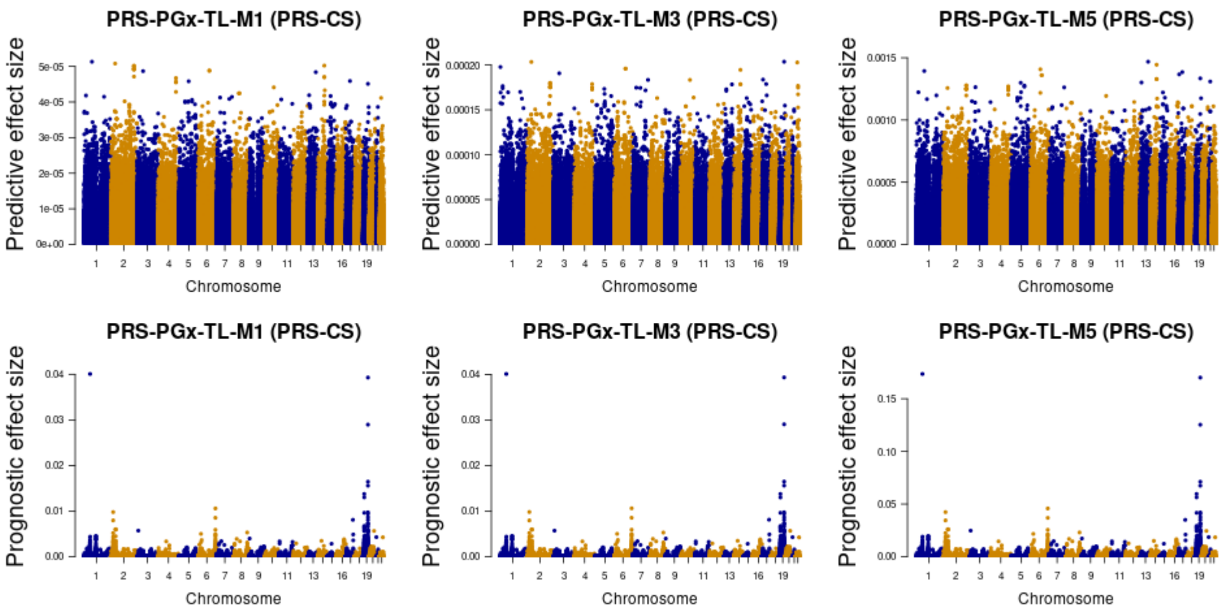

Figure S8 Manhattan plots of prognostic and predictive effect sizes for PRS-PGx-TL-M1/M3/M5 using PRS-CS as the baseline method from the PRS analysis of the IMPROVE-IT PGx GWAS data. The graph was created with the open-source qqman R package (https://github.com/stephenturner/qqman).

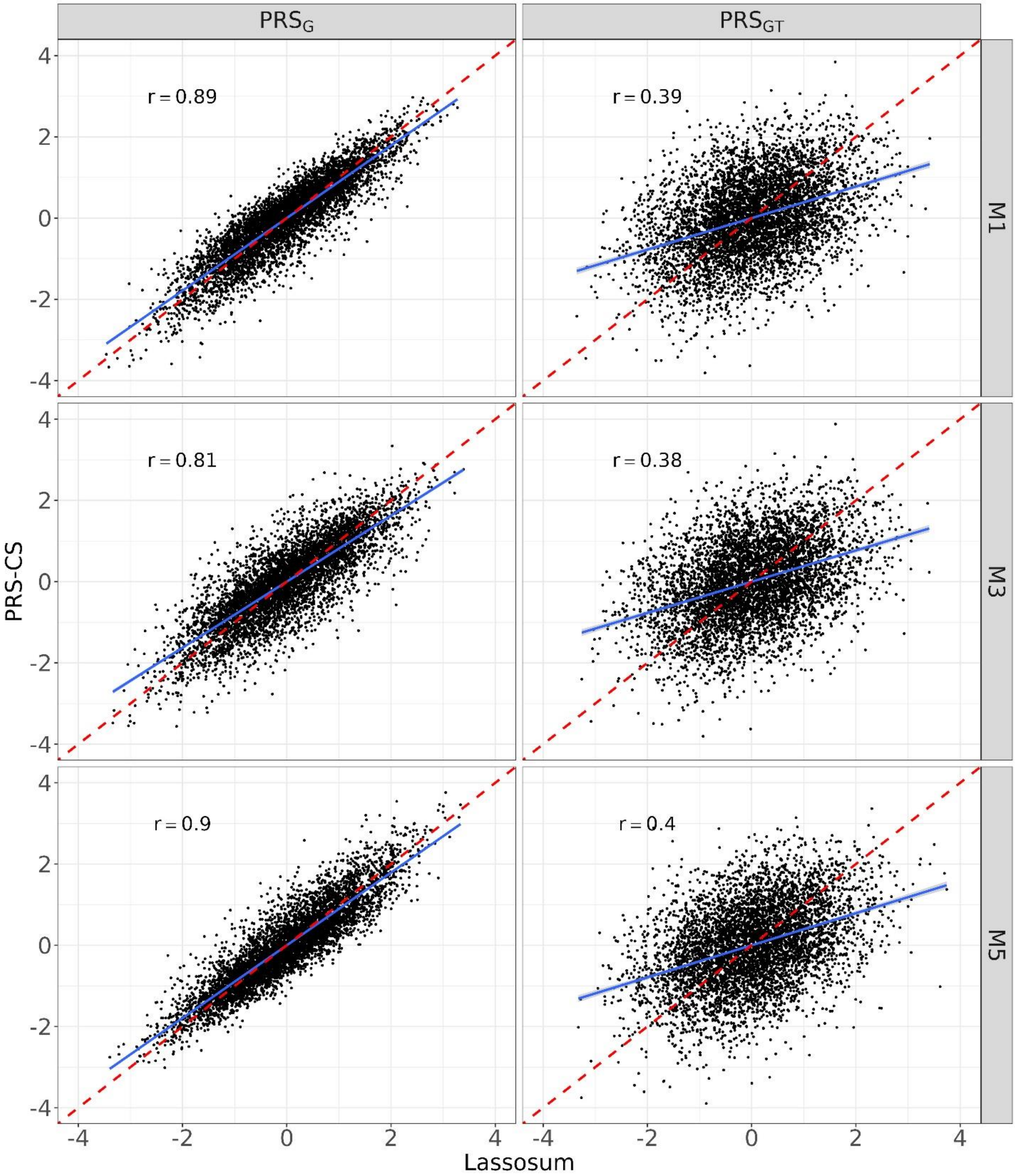

Figure S9 Scatter plots of PRS_G from Lassosum vs. PRS_G from PRS-CS and PRS_{GT} from PRS-CS vs. PRS_{GT} from PRS-CS for PRS-PGx-TL-M1, -M3 and -M5, respectively from the PRS analysis of the IMPROVE-IT PGx GWAS data. r stands for the Pearson's correlation. The graph was created with the open-source ggplot2 R package (https://ggplot2.tidyverse.org).

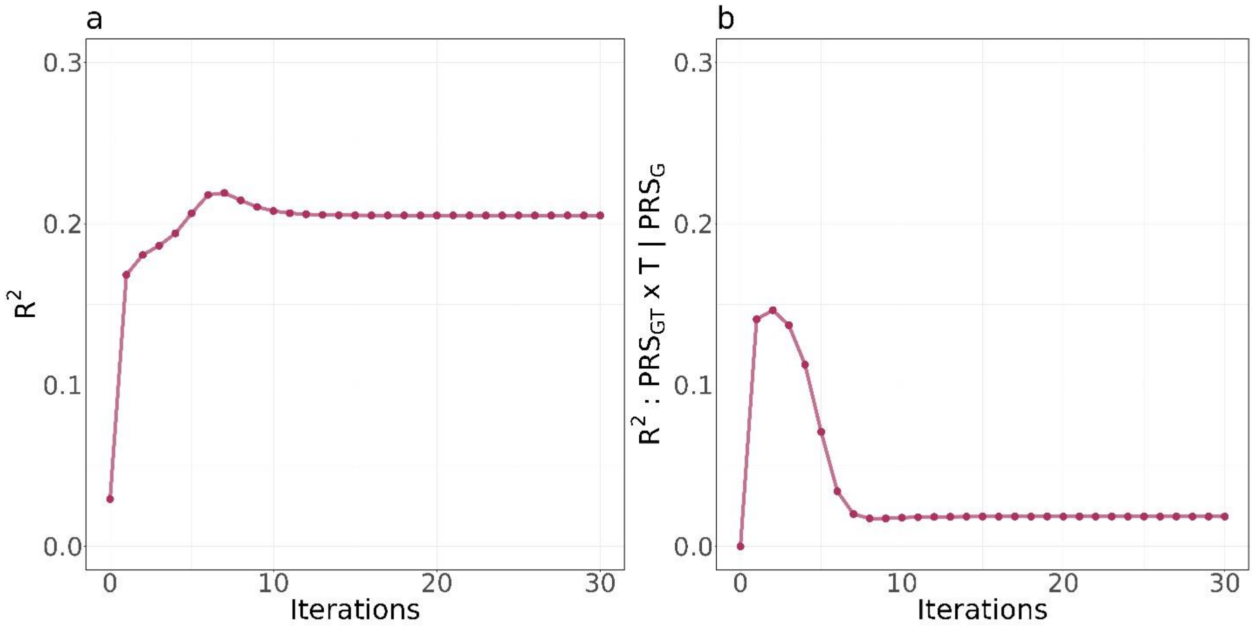

Figure S10 Accuracy change of the transfer learning method by number of iterations. Simulation result for (a) overall $\mathbf{R^2}$, (b) partial $\mathbf{R^2}$ explained by the $\mathbf{PRS_{GT}} \times \mathbf{T}$ term (conditional on $\mathbf{PRS_G}$). $\mathbf{H_D^2 = 0.3}$, $\boldsymbol{\gamma} = \mathbf{1}$, **pcausal = 0.01**, $\boldsymbol{\rho}_{\mathbf{DT}} = \mathbf{0.5}$, $\boldsymbol{\rho}_{\mathbf{E}} = \mathbf{0.5}$. The graph was created with the open-source ggplot2 R package (https://ggplot2.tidyverse.org).

Supplementary Tables

Table S1 Simulation parameters setup.

Parameter	Description	Value
p	Proportion of causal SNPs	0.1, 0.01, 0.001
h_T^2	Heritability to explain the trait (in the base cohort)	0.5
ρ_E	Correlation between prognostic and predictive effects in PGx GWAS	0.2, 0.5, 0.8
ρ_{DT}	Effect correlation between the trait in the base cohort and the drug response in the target cohort	0.2, 0.5, 0.8
G	IMPROVE-IT PGx GWAS genotype matrix with N patients and m SNPs	N = 5,000; m = 20,854 SNPs on chr 19
β_T	Coefficient of treatment	1
h_D^2	Heritability to explain the drug response (in the target cohort)	0.1, 0.3
γ	Scaling factor of predictive effect (so that prognostic and predictive effects may have different scales)	0.5, 1, 5

Table S2 Information about top 20 SNPs ranked by their absolute predictive effect sizes, estimated by PRS-PGx-TL-M1/M3/M5, respectively from the PRS analysis of the IMPROVE-IT PGx GWAS data. Lassosum was used as the baseline disease PRS method.

M1				M3				M5			
Chr	SNP	BP	Gene	Chr	SNP	BP	Gene	Chr	SNP	BP	Gene
19	rs7254892	45389596	*PVRL2*	19	rs7254892	45389596	*PVRL2*	19	rs7254892	45389596	*PVRL2*
19	rs7412	45412079	*APOE*	19	rs7412	45412079	*APOE*	7	rs1362234	75588366	*POR*
10	rs11196299	115073097	-	7	rs1362234	75588366	*POR*	10	rs11196299	115073097	-
2	rs2723147	23618209	*KLHL29*	10	rs11196299	115073097	-	19	rs7412	45412079	*APOE*
19	rs35918857	10254234	*DNMT1*	19	rs3208856	45296806	*CBLC*	19	rs35918857	10254234	*DNMT1*
7	rs1362234	75588366	*POR*	19	rs445925	45415640	*APOE*	9	rs11137198	140638534	*EHMT1*
19	rs3208856	45296806	*CBLC*	11	rs3136524	46750525	*F2*	16	rs210711	12611017	*SNX29*
6	rs6935708	29416652	*OR11A1*	2	rs2723147	23618209	*KLHL29*	13	rs3751432	41044128	*LINC00598*
16	rs210711	12611017	*SNX29*	6	rs6935708	29416652	*OR11A1*	1	rs11576216	179733859	*FAM163A*
13	rs4473067	44689605	*LINC00390*	19	rs35918857	10254234	*DNMT1*	5	rs7714291	111161642	*NREP*
11	rs3136524	46750525	*F2*	16	rs210711	12611017	*SNX29*	4	rs4610314	175240518	*CEP44*

19	rs445925	45415640	*APOE*	6	rs2281027	26446705	*BTN3A3*	4	rs17060463	175241378	*CEP44*
2	rs1530046	23623283	*KLHL29*	13	rs4473067	44689605	*LINC00390*	5	rs10043715	173594472	*NSG2*
2	rs2577747	23624756	*KLHL29*	2	rs1530046	23623283	*KLHL29*	1	rs12401944	109239079	*PRPF38B*
6	rs2281027	26446705	*BTN3A3*	2	rs2577747	23624756	*KLHL29*	11	rs7952632	95711345	*MAML2*
1	rs11576216	179733859	*FAM163A*	1	rs11576216	179733859	*FAM163A*	13	rs549431	75874682	*TBC1D4*
4	rs4610314	175240518	*CEP44*	16	rs17666927	72143414	*DHX38*	1	rs11591147	55505647	*PCSK9*
4	rs17060463	175241378	*CEP44*	13	rs3751432	41044128	*LINC00598*	22	rs5752079	25597842	*CRYBB3*
1	rs17112640	56161325	*RP11-466L17.1*	16	rs17666993	72146666	*DHX38*	6	rs11964205	163418400	*PACRG*
1	rs17112655	56168803	*RP11-466L17.1*	1	rs17112640	56161325	*RP11-466L17.1*	2	rs12621976	144106558	*ARHGAP15*

Table S3 Information about top 20 SNPs ranked by their absolute predictive effect sizes, estimated by PRS-PGx-TL-M1/M3/M5, respectively from the PRS analysis of the IMPROVE-IT PGx GWAS data. PRS-CS was used as the baseline disease PRS method.

M1				M3				M5			
Chr	SNP	BP	Gene	Chr	SNP	BP	Gene	Chr	SNP	BP	Gene
1	rs10493495	72722584	*NEGR1*	19	rs7254892	45389596	*PVRL2*	13	rs12429177	93071017	*GPC5*
2	rs17023938	39773237	*AC007246.3*	2	rs17023938	39773237	*AC007246.3*	14	rs759591	77551671	*RP11-7F17.4*
14	rs759591	77551671	*RP11-7F17.4*	22	rs5752851	29398459	*ZNRF3*	6	rs6932200	91787815	-
2	rs6754838	214375447	*SPAG16*	1	rs6662635	1220425	*SCNN1D*	1	rs10493495	72722584	*NEGR1*
2	rs16850465	214430651	*SPAG16*	6	rs9322774	104303973	-	17	rs9905211	37260188	*PLXDC1*
2	rs2130057	214249895	*SPAG16*	6	rs9322775	104304350	-	17	rs3935950	7974179	*ALOX12B*
2	rs1503380	214324201	*SPAG16*	14	rs759591	77551671	*RP11-7F17.4*	6	rs9322774	104303973	-
2	rs10498007	214345142	*SPAG16*	3	rs3732514	56667480	*FAM208A*	6	rs9322775	104304350	-
2	rs16850416	214354140	*SPAG16*	17	rs3935950	7974179	*ALOX12B*	19	rs7254892	45389596	*PVRL2*
2	rs6709713	214378285	*SPAG16*	7	rs8176059	142651354	*KEL*	14	rs2021766	77546692	*RP11-7F17.4*
6	rs9322774	104303973	-	10	rs12098564	86953327	*RP11-181F12.1*	14	rs204982	77553969	*RP11-7F17.4*

6	rs9322775	104304350	-	2	rs6754838	214375447	*SPAG16*	21	rs17811387	30413855	*USP16*
3	rs3732514	56667480	*FAM208A*	2	rs16850465	214430651	*SPAG16*	13	rs3809376	31308722	*ALOX5AP*
13	rs12429177	93071017	*GPC5*	4	rs3792614	164532973	*MARCH1*	5	rs157237	89389697	-
2	rs7587811	214457257	*SPAG16*	17	rs9905211	37260188	*PLXDC1*	2	rs6754838	214375447	*SPAG16*
14	rs2021766	77546692	*RP11-7F17.4*	14	rs2021766	77546692	*RP11-7F17.4*	2	rs16850465	214430651	*SPAG16*
14	rs204982	77553969	*RP11-7F17.4*	14	rs204982	77553969	*RP11-7F17.4*	5	rs7708937	123169574	-
4	rs3792614	164532973	*MARCH1*	13	rs12429177	93071017	*GPC5*	4	rs3792614	164532973	*MARCH1*
17	rs9905211	37260188	*PLXDC1*	2	rs2130057	214249895	*SPAG16*	5	rs10519734	123191183	-
5	rs1422174	92194417	-	22	rs8138016	36702149	*MYH9*	3	rs3732514	56667480	*FAM208A*

Table S4 Computation time for of PRS-PGx-TL with different baseline methods (Lassosum, PRS-CS) and different strategies (M1-M6) in both simulations and real (IMPROVE-IT GWAS) data analysis. In simulations, the mean and standard deviation (SD) of computation time was based on 1,000 repeats with the following parameters $\mathbf{H_D^2 = 0.3}$, $\boldsymbol{\gamma = 1}$, $\mathbf{pcausal = 0.01}$, $\boldsymbol{\rho_{DT} = 0.5}$, $\boldsymbol{\rho_E = 0.5}$. In real data analysis, parallel computing was used to compute the five folds of the "outer layer" and 22 chromosomes simultaneously.

**PRS method**	**Mean (SD) of time in simulations (minutes)**	**Time in real** (IMPROVE-IT GWAS) **data analysis (minutes)**
PRS-PGx-TL-M1 (Lassosum)	3.29 (2.28)	5.96
PRS-PGx-TL-M2 (Lassosum)	3.27 (2.26)	6.00
PRS-PGx-TL-M3 (Lassosum)	3.40 (2.39)	5.94
PRS-PGx-TL-M4 (Lassosum)	3.41 (2.45)	6.01
PRS-PGx-TL-M5 (Lassosum)	1.72 (0.73)	3.64
PRS-PGx-TL-M6 (Lassosum)	1.72 (0.72)	3.49
PRS-PGx-TL-M1 (PRS-CS)	17.31 (0.42)	62.36
PRS-PGx-TL-M2 (PRS-CS)	17.23 (0.49)	63.12
PRS-PGx-TL-M3 (PRS-CS)	17.25 (0.48)	62.16
PRS-PGx-TL-M4 (PRS-CS)	17.24 (0.33)	63.13
PRS-PGx-TL-M5 (PRS-CS)	9.09 (0.22)	33.62
PRS-PGx-TL-M6 (PRS-CS)	9.09 (0.25)	34.17

Table S5 A completed CONSORT checklist of IMPROVE-IT trial (registry name: ClinicalTrials.gov, registration number: NCT00202878, and the date of registration: September 13, 2005) is summarized in the following table, which is from the Cannon, C. P. *et al.* 2015 paper (Cannon, C. P. *et al.* Ezetimibe added to statin therapy after acute coronary syndromes. N. Engl. J. Med. 372, 2387–2397 (2015)).
